# Knee loading in OA subjects is correlated to flexion and adduction moments and to contact point locations

**DOI:** 10.1038/s41598-021-87978-2

**Published:** 2021-04-21

**Authors:** Ali Zeighami, Raphael Dumas, Rachid Aissaoui

**Affiliations:** 1 Laboratoire de Recherche en Imagerie et Orthopédie (LIO), Centre de Recherche du CHUM, École de Technologie Supérieure (ÉTS), Viger Tower, 900 St-Denis, Montreal, QC H2X0A9 Canada; 2 Univ Lyon, Univ Gustave Eiffel, LBMC UMR_T9406, 69622 Lyon, France

**Keywords:** Orthopaedics, Biomedical engineering

## Abstract

This study evaluated the association of contact point locations with the knee medial and lateral contact force (F_med_, F_lat_) alterations in OA and healthy subjects. A musculoskeletal model of the lower limb with subject-specific tibiofemoral contact point trajectories was used to estimate the F_med_ and F_lat_ in ten healthy and twelve OA subjects during treadmill gait. Regression analyses were performed to evaluate the correlation of the contact point locations, knee adduction moment (KAM), knee flexion moment (KFM), frontal plane alignment, and gait speed with the F_med_ and F_lat_. Medial contact point locations in the medial–lateral direction showed a poor correlation with the F_med_ in OA (R^2^ = 0.13, *p* = 0.01) and healthy (R^2^ = 0.24, *p* = 0.001) subjects. Anterior–posterior location of the contact points also showed a poor correlation with the F_med_ of OA subjects (R^2^ = 0.32, *p* < 0.001). Across all subjects, KAM and KFM remained the best predictors of the F_med_ and F_lat_, respectively (R^2^ between 0.62 and 0.69). Results suggest different mechanisms of contact force distribution in OA joints. The variations in the location of the contact points participate partially to explains the F_med_ variations in OA subjects together with the KFM and KAM.

## Introduction

Excessive or less frequent loading, misplaced contact regions, and altered muscle loading are among the biomechanical factors associated with the pathogenesis of knee osteoarthritis (OA)^1^. Knee OA is mostly developed on the medial compartment through which a major percentage of the total contact force (F_tot_) is transferred^1–3^. Reducing the knee medial contact force (F_med_) has been the focus of many studies using osteotomy surgery^4^, or noninvasive techniques such as cane or shoe soles, gait modifications, and valgus braces^5–8^ which could eventually change the frontal plane alignment or knee adduction moment (KAM). Therefore, characterizing the biomechanical parameters that contribute to the F_med_ can help to get insight the mechanism of OA initiation and help to find more effective therapeutic interventions to potentially slow down the OA progression.

Due to the complexity and the limited feasibility of estimating the contact forces using either musculoskeletal models or instrumented implants, the KAM has been widely used as a surrogate for the F_med_ or the medial-to-total contact force ratio (MR)^9–16^. Despite the considerable evidence on the relationship between the KAM with the F_med_^17^, and with the medial compartment OA progression^1,18,19^, the level of KAM correlation with the F_med_ is still debated^20^. In fact, the coefficient of determination (R^2^) between KAM and F_med_ from the linear regression models in previous studies falls in a wide range from 19 to 76%^14–16,21–23^. The values of R^2^ were also considerably variable across the subjects suggesting that the KAM is much less able to explain the variability in the F_med_ and/or MR in some individuals^14^. Several other parameters were suggested to increase the F_med_. Among those, the knee flexion moment (KFM)^23,24^, frontal plane alignment^20,25^, and gait speed^14^ were found to have substantial correlation with the F_med_. Moreover, sensitivity analysis studies showed that the anterior–posterior and medial–lateral locations of the tibiofemoral contact points had also a high impact on the contact forces and the force distribution between the medial and lateral compartments^26–29^. For instance, in a simulation study, Lerner et al. (2015) reported a 6% increase in the MR per each millimeter of the contact point medial shift (R^2^ = 0.99). Since there has been no means to straightforwardly incorporate the subject-specific contact point trajectories into the contact force estimations, no study could have tested the association of contact point locations with the knee medial and lateral contact force. Recently, Zeighami et al.(2018)^28^ incorporated the subject-specific contact point trajectories obtained from 3D/2D registration techniques into the medial and lateral knee contact force estimations. This technique currently allows evaluating the correlation of the contact point locations with the F_med_ and F_lat_ alongside the other parameters.

In addition, most of the studies on the F_med_ are performed in knee arthroplasty subjects, and the data on the intact healthy and OA knees are scarce. The few available OA-control studies which estimated the F_med_ and F_lat_ used a classical linear model of the contact point trajectories or a generic deformable model of the knee^24,30–34^, and therefore, did not take into account the inter-subject variations and the distinct patterns of the anterior–posterior and medial–lateral contact point locations in healthy and OA subjects^35,36^.

Therefore the objectives of this study are (1) to estimate the F_med_ and F_lat_ in both healthy and OA subjects using the subject-specific contact point trajectories obtained from 3D/2D registration techniques, and (2) to analyze the impact of the medial–lateral and anterior–posterior contact point locations along with KAM, KFM, gait speed, and frontal plane alignment on the F_med_ and F_lat_.

## Materials and methods

### Experimental protocol

Ten healthy (6 men, 4 women, 55 yrs., 1.68 m, 71 kg) and 12 severe OA (2 men, 10 women, 59 yrs.1.61 m, 85.53 kg, K–L grade 4) subjects were asked to walk at their comfortable speed on an instrumented split-belt treadmill for 45 s (Table 1). Data from force platforms and reflective markers mounted on the kneeKG™ system^37^ were filtered using a zero-lag 2nd order Butterworth filter with cut-off frequencies automatically calculated using a power spectrum analysis (PSA) algorithm^38^.

**Table 1:** The anthropometrics, frontal plane alignment and gait speed of 10 healthy (H) and 12 OA subjects.

Subject	Height (m)	Gender	Weight (kg)	Age (yrs)	BMI	Frontal plane alignment (deg)	Gait speed (m/s)
**Healthy subjects**							
H01	1.73	M	76.9	39	25.69	1.91	0.96
H02	1.5	M	54	66	24	− 2	0.46
H03	1.71	M	84.5	38	28.9	0.98	0.67
H04	1.66	F	58.1	57	21.08	3.63	0.9
H05	1.81	M	81.9	61	25	− 1.59	0.82
H06	1.64	F	60.8	60	22.61	− 1.76	0.79
H07	1.73	M	89.8	61	30	1.67	0.57
H08	1.56	F	58.3	60	23.96	− 0.96	0.45
H09	1.75	M	80.7	59	26.35	6.75	0.42
H10	1.75	F	60.6	53	19.79	− 2.97	0.48
Average (SD)	1.68 ± 0.1		70.56 ± 13.38	55.40 ± 9.49	24.74 ± 3.20	0.57 ± 3.02	0.65 ± 0.20
**OA subjects**							
OA01	1.64	F	99.34	56	36.93	0.5	0.54
OA02	1.63	F	85.8	61	32.29	3.6	0.77
OA03	1.75	M	87	66	28.41	6.8	0.71
OA04	1.61	F	95	56	36.65	9.8	0.5
OA05	1.5	F	74	53	32.89	7.8	0.51
OA06	1.63	F	81.6	52	30.71	6.95	0.49
OA07	1.72	M	84	69	28.39	7.75	0.49
OA08	1.63	F	91.8	58	34.55	10.03	0.34
OA09	1.67	F	98.4	57	35.28	10.98	0.4
OA10	1.55	F	72.57	62	30.21	− 2.3	0.4
OA11	1.58	F	74.4	64	29.8	6.61	0.31
OA12	1.52	F	82.5	61	35.71	6.65	0.48
Average (SD)	1.62 (0.1)		85.53 ± 9.2*	59.58 ± 5.2	32.65 ± 3.1*	6.26 ± 3.9 *	0.5 ± 0.1

Average data ± 1 SD are provided; * denotes a statistically significant difference from the healthy group.

**Figure 1:**
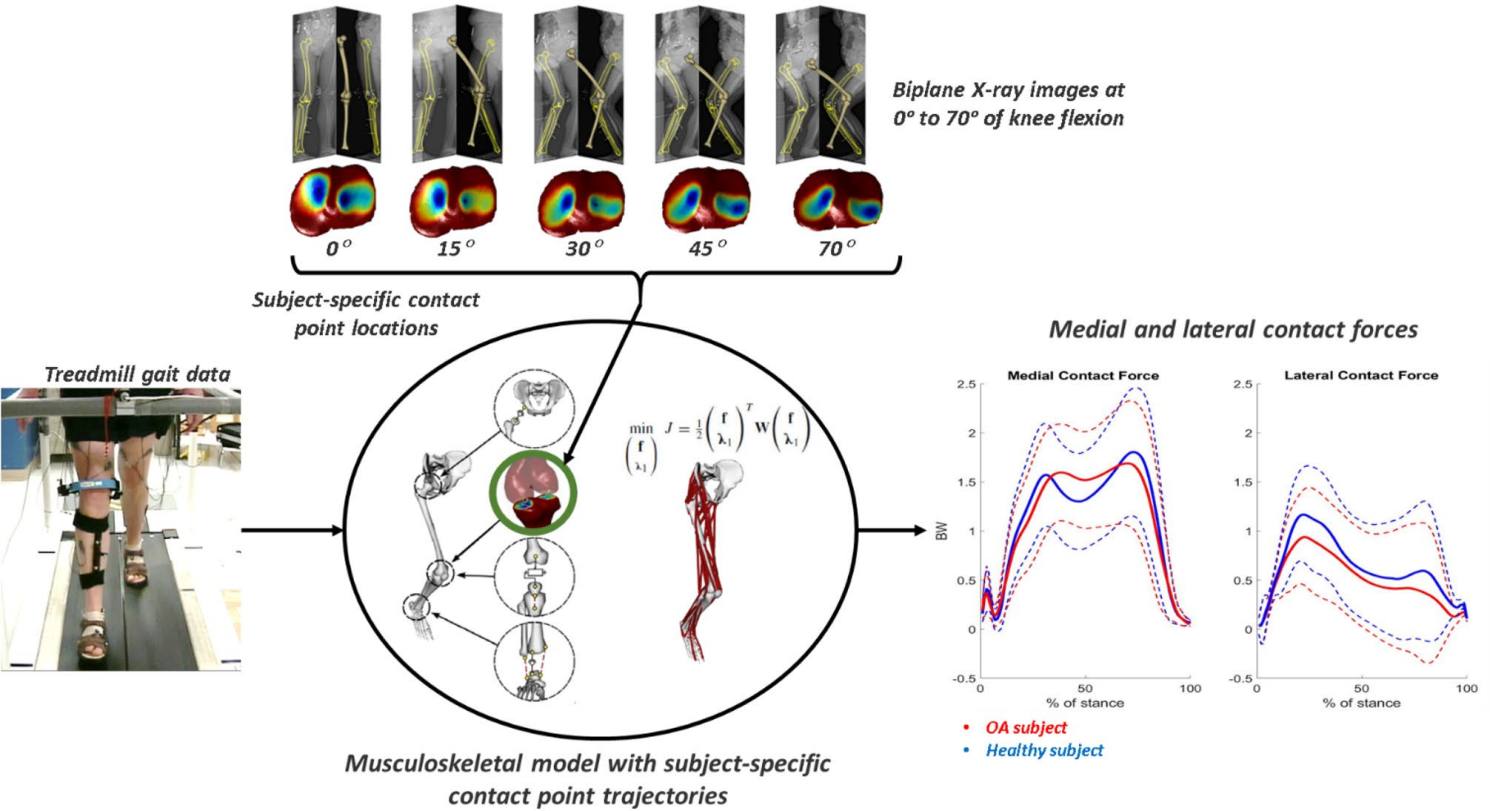
The process of estimating the medial and lateral contact forces using the subject-specific contact point trajectories.

The subject-specific contact point trajectories were approximated using a weighted center of bone-to-bone proximity algorithm during a quasi-static squat task^36^. The 3D models of the tibia and femur were reconstructed and registered from EOS™ low-dose biplane X-ray images of the subjects recorded at 0°, 15°, 30°, 45°, and 70° of knee flexion (Fig. 1). The subject-specific tibiofemoral contact point trajectories were built as a function of the knee flexion angle as described earlier^28^. The frontal plane alignment was measured from the reconstructed tibia and femur of the subjects at the standing posture (~ 0°).

All subjects signed an informed consent form and the experimental protocol was approved by the ethics committees of the Centre de Recherche, Centre Hospitalier de l’Université de Montréal (CRCHUM) and École de Technologie Supérieure de Montréal (ÉTS). All the research and methods in this study were performed in accordance with the CRCHUM and ÉTS ethics committee guidelines as well as with the Helsinki Declaration of 1975, as revised in 2000.

### Musculoskeletal model with subject-specific contact point trajectories

The medial and lateral knee contact forces were estimated using a musculoskeletal model of the lower limb with the integration of the subject-specific contact point trajectories^28^ (Fig. 1). The model consists of 5 segments and 5 joint degrees of freedom (DOF), with the hip joint modeled as a ball and socket joint (3 DOF) and the ankle modeled as a ball and socket joint plus two isometric ligaments (1 DOF). The tibiofemoral joint (1 DOF) is defined by 5 kinematic constraints derived from the subject-specific tibiofemoral contact point trajectories: at each flexion angle, the femoral and tibial contact points are superimposed in the 3 spatial directions on the medial compartment, and in both anterior–posterior and proximal–distal directions on the lateral compartment. The proximal–distal constraints on each compartment allow for a straightforward computation of medial and lateral contact forces^28^. The musculo-tendon origin and insertion points were adopted from Delp et al. (1990)^39^.

A full description of the musculoskeletal model of the lower limb (except for the tibiofemoral joint) is detailed in^40^. The model medial and lateral contact force estimations, without subject-specific contact point trajectories, were previously validated^40–42^ against instrumented implants data. For a semi-quantitative validation of the model with the subject-specific tibiofemoral contact points, the active/inactive state of 8 muscles was compared (i.e. concordance coefficients^43^) to the EMG signals to check if the model predictions are realistic for 10 healthy subjects^28^. The EMG concordance coefficients for the OA and healthy subjects of the current study are provided in the supplementary material (1).

The contact forces were calculated in a one-step procedure simultaneously minimizing the contact and musculo-tendon forces. Internal joint moments are computed by inverse dynamics (they equal the inter-segmental action of muscles, ligaments, and contacts forces in the model) and represent the action of the proximal onto the distal segment expressed in the joint coordinate system^44–46^. The external knee adduction (KAM) and knee flexion moments (KFM) were obtained by reversing the corresponding internal moment signs.

The knee contact forces were normalized to body weight (BW) and the KAM and KFM were normalized to BW*height.

### Statistical analysis

Linear regression tests were performed to evaluate the extent to which the independent variables were predictive of the F_med_ and F_lat_ in OA and healthy subjects. Given the limited number of subjects, the analyses were limited to simple linear regressions only. The dependent variables were F_med_ and F_lat_ at 4 peak instances being the 1st and 2nd medial and lateral peaks. The independent variables were the KAM, KFM, frontal plane alignment, gait speed, and positions of the medial and lateral contact points in the anterior–posterior (CPxmed, CPxlat), and medial–lateral (CPzmed, CPzlat) directions at the corresponding timing. The rationale for considering 4 peaks was because the peaks occurs at different timings of the medial and lateral contact. A non-parametric Mann–Whitney U-test was performed to compare all independent and dependent variables between the OA and healthy subjects (*p* < 0.05). The coefficients of determination (R^2^) were compared to identify the parameters that explains the greatest proportion of the variance of the dependent variables. The correlation was considered poor, moderate, or good if R^2^ ≤ 0.50, 0.50 < R^2^ < 0.75, and R^2^ > 0.75, respectively. The regression model was rated as significant for *p* < 0.05. The correlation with each variable was independently tested.

## Results

### OA and healthy group comparison

**Figure 2:**
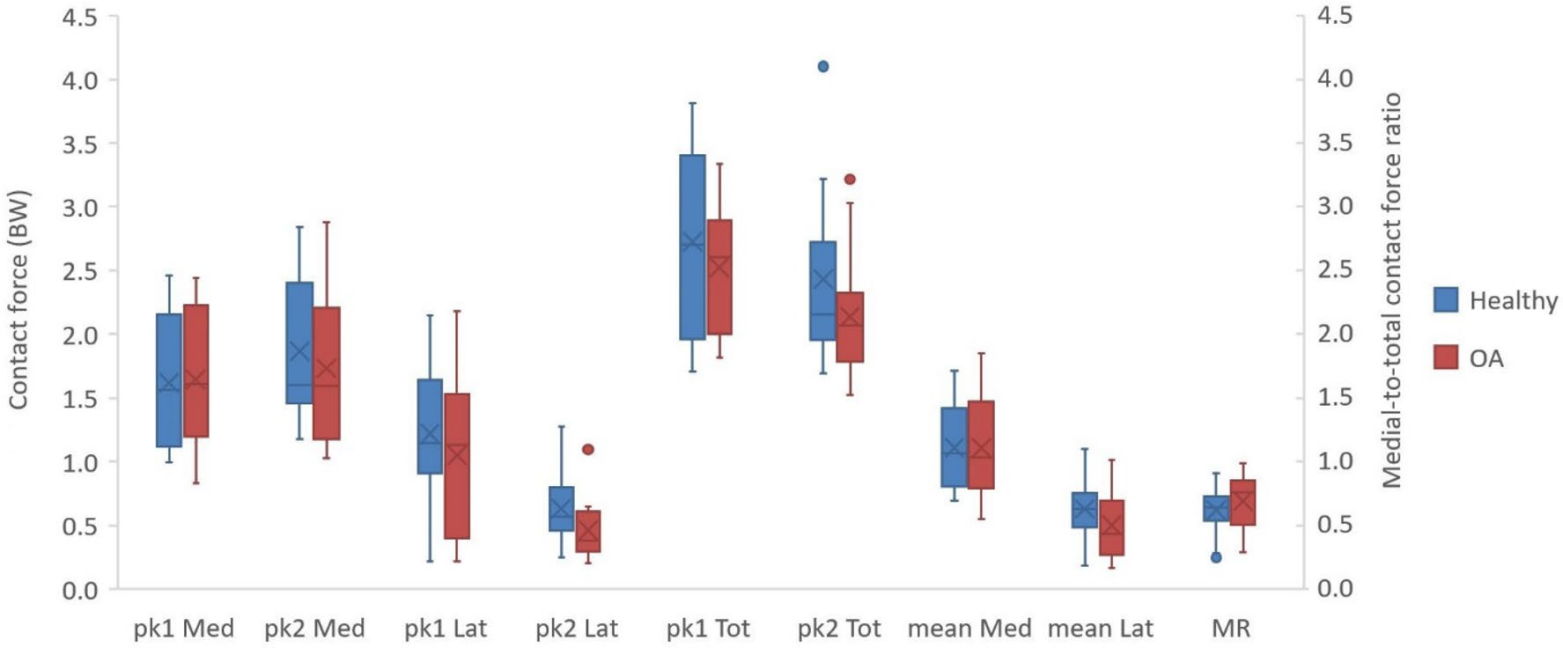
Box and whisker plot of contact forces of the healthy (blue) and OA (red) groups at the 1st and 2nd peaks of medial contact force (pk1 Med, pk2 Med), 1st and 2nd peaks of the lateral contact force (pk1 Lat, pk2 Lat), 1st and 2nd peaks of the total contact force (pk1 Tot, pk2 Tot), the average medial (mean Med) and lateral (mean Lat) contact forces during the stance phase, and the medial-to-total contact force ratio (MR) during the stance phase. The plot represents the minimum, maximum, lower and higher quartiles, and the median as well as the mean value (X mark), and the outliers (o mark).

The differences at 1st and 2nd peak F_med_, F_lat_, and F_tot_ were not significant between the OA and healthy groups (*p* > 0.05) (Fig. 2). The contact forces in healthy and OA subjects averaged slightly higher than the OA subjects at the 2nd peak F_med_ (OA = 1.7 BW, healthy = 1.9 BW), 1st peak F_lat_ (OA = 1.1 BW, healthy = 1.2 BW), 2nd peak F_lat_ (OA = 0.5 BW, healthy = 0.6 BW), 1st peak F_tot_ (OA = 2.5 BW, healthy = 2.7 BW), and 2nd peak F_tot_ (OA = 2.1 BW, healthy = 2.4 BW), and were similar at the 1st peak F_med_ (OA = healthy = 1.6 BW). The F_med_, F_lat_, and F_tot_ over the stance phase are presented in the supplementary material (2).

The average KAM in OA subjects at the timing of the 1st and 2nd peaks of F_med_ and F_lat_ were significantly greater than that of the healthy subjects (*p* < 0.05). Contra wise, the average KFM was not significantly different between OA and healthy subjects (*p* > 0.05). The KAM and KFM plots are provided in the supplementary material (3).

The CPzmed and CPzlat at the timing of the 1st and 2nd peaks of F_med_ and F_lat_ represented significant differences (*p* < 0.001) between the two groups while the CPxmed and CPxlat were not significantly different (*p* > 0.05). The individual contact point trajectories are provided in the supplementary material (4) and were extensively described in Zeighami et al. (2017)^36^.

The frontal plane alignment in OA subjects (6.26°) was significantly greater than in the healthy subjects (0.57°, *p* < 0.01). The groups were not matched for height, BMI (*p* < 0.05), and gender (Table 1). Gait speed did not significantly differ between the two groups (*p* = 0.11).

### Medial and lateral contact force (F_med,_ F_lat_) regression

The linear regression tests revealed that the KAM was the best predictor of F_med_ both for OA (R^2^ = 0.62) and healthy (R^2^ = 0.62) subjects. No other variable accounted for more than 50% of the variance in F_med_ in either group (Table 2). CPxmed was the second-best predictor of F_med_ in OA subjects (R^2^ = 0.32) followed by the KFM (R^2^ = 0.16), gait speed (R^2^ = 0.15), and CPzmed (R^2^ = 0.13). In healthy subjects, the gait speed (R^2^ = 0.27) and CPzmed (R^2^ = 0.24) had the strongest correlation with the F_med_ after the KAM.

**Table 2:** Simple regressions models in OA and healthy groups with F_med_ (a) and F_lat_ (b) as dependent variables.

OA/healthy	Dep. variable	Indep. variable	R^2^	adj. R^2^	p (model sig.)	c1 (y intcp.)	c2
**(a) Medial contact force regression**							
OA	Fmed	KAM	0.62	0.61	0.000	1.12	0.48
OA	Fmed	KFM	0.16	0.14	0.005	1.65	− 0.13
OA	Fmed	CPzmed	0.13	0.12	0.010	2.76	0.07
OA	Fmed	CPxmed	0.32	0.31	0.000	0.07	0.14
OA	Fmed	Frontal plane alignment	0.04	0.02	0.196	1.71	− 0.03
OA	Fmed	Gait Speed	0.15	0.13	0.007	0.58	1.87
Healthy	Fmed	KAM	0.62	0.61	0.000	1.68	0.62
Healthy	Fmed	KFM	0.01	− 0.02	0.597	1.60	− 0.03
Healthy	Fmed	CPzmed	0.24	0.22	0.001	3.31	0.13
Healthy	Fmed	CPxmed	0.04	0.02	0.201	1.19	0.04
Healthy	Fmed	Frontal plane alignment	0.01	− 0.02	0.550	1.54	0.02
Healthy	Fmed	Gait Speed	0.27	0.25	0.001	0.39	1.79
**(b) Lateral contact force regression**							
OA	Flat	KAM	0.19	0.17	0.002	0.85	− 0.22
OA	Flat	KFM	0.69	0.68	0.000	0.44	0.22
OA	Flat	CPzlat	0.05	0.02	0.147	0.06	0.04
OA	Flat	CPxlat	0.10	0.08	0.032	0.80	− 0.05
OA	Flat	Frontal plane alignment	0.00	− 0.02	0.723	0.72	− 0.01
OA	Flat	Gait Speed	0.00	− 0.02	0.936	0.70	− 0.05
Healthy	Flat	KAM	0.05	0.02	0.166	0.83	− 0.13
Healthy	Flat	KFM	0.68	0.67	0.000	0.56	0.23
Healthy	Flat	CPzlat	0.06	0.04	0.124	0.33	0.03
Healthy	Flat	CPxlat	0.03	0.01	0.268	0.90	− 0.02
Healthy	Flat	Frontal plane alignment	0.01	− 0.02	0.590	0.87	− 0.01
Healthy	Flat	Gait Speed	0.16	0.13	0.012	0.20	1.01

The corresponding independent variable (indep. variable), (adjusted) coefficient of determination (adj.) R^2^, model significance p, and the regression coefficient corresponding to the independent variables and the y-intercepts (y intcp.) are given in the table. Independent variables consist of the following parameters: Knee adduction moment (KAM), knee flexion moment (KFM) and medial and lateral contact point locations in the anterior–posterior (CPxmed, CPxlat), and medial–lateral (CPzmed, CPzlat) directions, frontal plane alignment, and gait speed.

The KAM, CPzmed, and gait speed were significantly correlated to the F_med_ both in OA and healthy subjects. However, the F_med_ in healthy subjects increases faster due to a unit increase in the KAM and CPzmed as the slopes of the regressions in healthy subjects (KAM:c_2_ = 0.62, CPzmed:c_2_ = 0.13) are higher than those in OA subjects (KAM:c_2_ = 0.48, CPzmed:c_2_ = 0.07) (Table 2). The gait speed slopes were similar in OA and healthy subjects (Table 2).

The KFM was the dominant predictor of the F_lat_ in OA (R^2^ = 0.69) and healthy subjects (R^2^ = 0.68). Other significant parameters associated with the F_lat_ consists of KAM (R^2^ = 0.19) and CPxlat (R^2^ = 0.10) in OA subjects, and gait speed (R^2^ = 0.16) in healthy subjects.

The KAM slopes are positive in the F_med_ regressions both in OA and healthy subjects (OA KAM:c_2_ = 0.48, Healthy KAM:c_2_ = 0.62) while they are negative in the F_lat_ regressions (OA KAM:c_2_ = − 0.22, Healthy KAM:c_2_ = − 0.13). Therefore, an increase in the KAM is associated to increase in the F_med_ and a decrease in the F_lat_ in both groups.

## Discussion

The objective of this study was to estimate the knee contact forces in both healthy and OA subjects and to analyze the association of contact point locations with the knee medial and lateral contact force, alongside other parameters (KAM, KFM, frontal plane alignment, and gait speed). For that, we used a musculoskeletal model with subject-specific tibiofemoral joint contact point trajectories to investigate if the subject-specific contact point trajectories are correlated to the knee contact forces in OA and healthy subjects.

A large body of the literature reported the contact forces using prosthetic measurements or musculoskeletal model estimations. Our contact force estimations (Table 3) falls within the literature range for the peaks of F_med_ (2.1 ± 0.5 BW), F_lat_ (0.9 ± 0.4 BW), and F_tot_ (3.1 ± 0.8 BW)^6–8,11,27,30,40,47–53^. More recently, there has been an increased interest towards comparing the F_med_ and F_lat_ in OA and healthy subjects^24,30–32,34^. Sritharan et al. (2017)^30^ and Kumar et al. (2013)^24^ used musculoskeletal models with classical linear contact point trajectories to estimate the contact forces. They reported that despite the differences between the absolute values of the contact forces in the two groups (Table 3), the differences were not significant which is in accordance with our results. The peaks of F_med_ in both studies were greater than the peaks of F_lat_. Other studies reported similar values with slightly different contact forces between OA and healthy groups (Table 3). While we found no significant differences in the knee contact forces between OA and healthy subjects, the external moments, the contact point locations, and the corresponding regression coefficients and slopes were different, suggesting altered mechanisms of contact force distribution in the OA joint.

**Table 3:** The 1st and 2nd peaks of medial contact force (pk1–pk2 Med), 1st and 2nd peaks of the lateral contact force (pk1–pk2 Lat), and 1st and 2nd peaks of the total contact force (pk1–pk2 Tot) in the case-control studies with OA and healthy subjects.

Study	OA status	Number of subjects	CF estimation	Fmed		Flat		Ftot	
				Peak 1	Peak 2	Peak 1	Peak 2	Peak 1	Peak 2
Van Rossom et al.^34^	Healthy	19	MSK model	1.8	1.9	1.3	1	3.9	2.8
	OA medial	8		1.9	1.8	1.1	0.8	3	2.5
	OA lateral	7		1.5	1.6	1.2	1	2.6	2.5
Sritharan et al.^30^	Healthy	19	MSK model	2	3	0.3	0.52	2.3	3.5
	OA	39		2.1	3.3	0.3	0.59	2.3	3.8
Marouane et al.^60^	Healthy	1	FE model	3.2	3	2.6	0.8	4.1	3.7
	Simulated OA	1		2.7	3	1.8	0.7	3.3	3.5
Kumar et al.^24^	Healthy	16	MSK model	2.4	1.8	1.3	0.5	3.7	2.2
	OA	12		2.6	2.1	0.9	0.1	3.5	2.2
This study	Healthy	10	MSK model	1.6	1.9	1.2	0.6	2.7	2.4
	OA	12		1.6	1.7	1.1	0.5	2.5	2.1

The contact force (CF) estimations (BW) were obtained using musculoskeletal (MSK) or finite element (FE) models.

The external moments on the knee joint are thought to be counterbalanced by the musculotendon and the tibiofemoral contact forces. The static equilibrium of the knee joint in the frontal plane requires that the contact point locations be related to the F_med_ and F_lat_^54^. Therefore, it is conceivable that the load taken by each compartment is proportional to the contact point distance from the joint center as postulated by previous sensitivity analyses^27,29^. Nevertheless, a multifactorial study, considering the 3D joint equilibrium, suggested that there are other factors which confound a strong correlation between the contact point location and the F_med_^28^. However, the contributing parameters and their association with the F_med_ and F_lat_ modification were not previously investigated.

Overall, the F_med_ was better predicted by the contact point locations than the F_lat_ both in OA and healthy subjects. The contact point locations in the two directions (R^2^ = 0.13 and R^2^ = 0.32) and the contact point location in medial–lateral direction (R^2^ = 0.24) were among the significant, yet low, predictors of the F_med_ in OA and healthy subjects. A weak correlation between the peaks of F_med_ and the CPzmed was similarly reported in a previous study on healthy subjects^28^. The lateral contact point location in anterior–posterior (CPxlat) direction was the only component of the contact point slightly correlated with the F_lat_ in OA subjects (R^2^ = 0.10, *p* = 0.032). To our knowledge, our study is the first one to analyze the correlations between subject-specific contact point locations and contact forces. In the literature, sensitivity analyses have previously studied the impact of the contact point locations in both the anterior–posterior and medial–lateral directions and have established they are sensitive model parameters^26–29^. The reported sensitivity of 0.04 and 0.03 BW/mm on the first and second peaks of F_med_ are close to the slopes of regression found in our study^29^.

Before our study, it was not clear, especially in OA patients, how these correlations between the contact point location and the contact force compare with respect to other correlations. The KFM and KAM accounted for a high proportion of the variance in F_med_ and F_lat_ compared to the components of the contact point location. The KAM was the most powerful predictor of the F_med_ in both OA and healthy groups (R^2^ = 0.62), whereas it had a smaller effect on the F_lat_ only in the OA group (R^2^ = 0.19). The most powerful predictor of the F_lat_ was the KFM in both groups reflecting the contrasting influence of the sagittal and frontal plane knee moments on the medial and lateral contact forces.

The importance of the KFM in estimating the magnitude or variation of F_med_ was also highlighted in previous studies both in OA and healthy subjects^24,55^. Meireles et al. (2016)^56^ performed regression tests with KFM and KAM inputted independently. They found that in early OA subjects, the correlation of KFM and KAM with the 1^st^ peak of medial contact force were similar (R^2^ = 0.62, 0.67 accordingly). They also reported that, in healthy controls, the correlation with the KAM (R^2^ = 0.65) was much higher than with the KFM (R^2^ = 0.21). This implies the knee load sharing can be affected by the knee pathology with KFM having a more important impact on the F_med_ in OA group with respect to the healthy group. This is similar to the higher correlation of KFM with the F_med_ in OA subjects in our study. Manal et al. (2013)^57^ reported that the KFM adds an additional 22% to the 63% of the variance in the F_med_ peak which is explained by the KAM. Both moments together accounted for ~ 85% of the F_med_ variation. In the current study, we found a similar correlation of KAM (R^2^ ~ 0.62) with the F_med_. In an instrumented implant study, Trepczynski et al. (2014)^17^ observed that the KAM alone accounted for 87% of the F_med_ variation. The higher contribution of KAM in their study could be attributed to the prosthetic knee which has a different joint configuration and congruence compared to the native knees.

We did not find a significant correlation between the frontal plane alignment and the F_med_, or F_lat_ (*p* > 0.05). In an FE simulation study with one subject, the frontal plane alignment was reported a much more effective factor in reducing F_med_ compared to the KAM^20^. Nevertheless, Kumar et al. (2013)^24^ failed to find a significant correlation between frontal plane alignment and the F_med_ peak in neither healthy (n = 12) or OA (n = 16) groups. In our study, we did not find a significant correlation between the frontal plane alignments (at the standing posture) and F_med_ (*p* > 0.05).

Gait speed was correlated to the F_med_ and F_lat_ in healthy, and to the F_med_ in OA subjects. The F_med_ was similarily increases by a similar rate in OA and healthy subjects due to an increase in the gait speed as its slope of regression in both groups is similar.

The current study has several limitations. The subject-specific contact point trajectories were measured during a quasi-static squat and may vary in different weight-bearing tasks. Even though, Gasparutto et al. (2015)^58^ showed that the impact of the dynamic activity on the couplings between the joint degrees of freedom was limited. Similarly, a recent systematic review^59^ showed that CPxmed and CPxlat were in the range of other contact point trajectories measured by fluoroscopy on both OA and healthy subjects during various weight-bearing tasks (gait, step-up, kneeling, squat…). The estimation of the contact point trajectories is based on a weighted center of bone-to-bone proximity^28^ and is subject to inaccuracies in approximating the center of pressure. This is primarily due to the absence of cartilage layer and menisci in the X-ray images and the errors in the reconstruction and registration process^36,60,61^. Moreover, the contact point trajectories were interpolated from a limited number of contact point locations. Finally, the number of subjects is not enough to generalize the conclusions made in this study.

As a conclusion, in the current work, we assessed the association of the contact point locations with the alterations of the knee contact forces and their distribution. F_med_ was influenced more than the F_lat_ by the contact point locations in both directions especially in OA subjects. Overall, the contact point locations had lower correlation with respect to the KFM and KAM and the two moments remained the best predictors of the F_med_ and F_lat_. KAM and KFM can be easily estimated from classical inverse dynamics while obtaining subject-specific contact points require more complex procedures. However, the correlation between the contact points and the contact forces helps to understand the proportion of the variation in the contact forces which is not explained by the KAM and KFM. Even though the contact force variables (F_med_, F_lat_, and F_tot_) did not present significant differences between the OA and healthy groups, the correlations were different (typically the slopes of regression), suggesting altered mechanisms of contact force distribution in the OA joint. Knowledge of the association of various parameters with the knee contact forces distribution could eventually lead to better understand the OA progression mechanism and help better planning the most effective interventions to slow the disease process. In a review of the biomechanical characteristics that have possible influence over articular tissue loading in OA, altered KAM and KFM, as well as slower gait speed (together with more flexed knees, reduced range of motion, muscle atrophy and other characteristics that were not analyzed in our study) were associated to lower contact forces, altered distribution of F_med_ and F_lat_ and different region of articular surface loaded^62^. The altered contact point trajectories were not directly listed in this review and our study demonstrate that this characteristics is also associated to altered distribution of F_med_ and F_lat_. Typically, a more posterior and medial contact point location in the medial compartment is associated to a lower F_med_ in OA patients.

## Supplementary Information

Supplementary Information 1.

Supplementary Information 2.

Supplementary Information 3.

Supplementary Information 4.

## Data Availability

All data generated or analyzed during this study are included in this published article (and its Supplementary Information files).

## Abbreviations

OA: Knee osteoarthritis
F_tot_: Knee total contact force
F_med_: Knee medial contact force
F_lat_: Knee lateral contact force
MR: Medial-to-total contact force ratio
KAM: Knee adduction moment
KFM: Knee flexion moment
R^2^: Coefficient of determination
DOF: Degrees of freedom
BW: Body weight
CPzmed: Medial contact point in the anterior–posterior direction
CPzlat: Lateral contact point in the anterior–posterior direction
CPxmed: Medial contact point in the medial–lateral direction
CPxlat: Lateral contact point in the medial–lateral direction
