# Supplementary material for: Knee loading in OA subjects is correlated to flexion and adduction moments and to contact point locations
Source: Sci Rep. 2021 Apr 21;11:8594. doi: 10.1038/s41598-021-87978-2 (PMC8060429; doi:10.1038/s41598-021-87978-2)
Supplement: Supplementary file 1 — Supplementary Information 1. [file 41598_2021_87978_MOESM1_ESM.docx]

Knee loading in OA subjects is correlated to flexion and adduction moments and to contact point locations

Ali Zeighami^1^, Raphael Dumas^2^, Rachid Aissaoui^1*^

1. Laboratoire de Recherche en Imagerie et Orthopédie (LIO), École de Technologie Supérieure (ÉTS), Centre de Recherche du CHUM, Montréal, Québec, Canada
2. Univ Lyon, Univ Gustave Eiffel, LBMC UMR_T9406, F69622, Lyon, France

**Supplementary material 1:**

**Musculo-tendon forces semi-quantitative validation using EMG data**

**Musculo-tendon forces semi-quantitative validation using EMG data**

The surface electromyography (EMG) signals of 10 healthy and 12 OA subjects were compared to the musculo-tendon activity from the musculoskeletal (MSK) model to determine if the model estimations are realistic.

EMG signals of the rectus femoris (RF), vastus lateralis (VL), vastus medialis (VM), tibialis anterior (TA), gastrocnemius medialis (GM), gastrocnemius lateralis (GL), semitendinosus (ST), and biceps femoris (BF) muscles were collected at 2 kHz during the trials using a Delsys Myomonitor system (Delsys, Inc., Boston, MA). A semi-quantitative validation of estimated musculo-tendon forces from the MSK model was performed to find the active/inactive concordance with the EMG signals [^1^](#_ENREF_1). Only stance phase is analyzed. The stance phase was divided into 4 sub-phases (i.e., loading response, mid-stance, terminal stance, pre-swing) during which the EMG envelope and the musculo-tendon force states could take active or inactive state. Muscles were defined active when the mean value during a phase was above 20% of the maximum of the estimated musculo-tendon force and of the EMG envelope, respectively [^2^](#_ENREF_2). The concordance coefficient represents the ratio of phases that indicate concordance (i.e., simultaneously active or inactive) during the gait trial.

**Results**

The average EMG coefficients of concordance showed no remarkable difference between the OA and healthy subjects. The VM and VL muscles demonstrated the best concordance in both groups where the GM and GL showed the lowest coefficients of concordance (Table S4-1).

*Table S1-1: Coefficient of concordance (%) in OA (a) and healthy (b) subjects for the rectus femoris (RF), vastus lateralis (VL), vastus medialis (VM), tibialis anterior (TA), gastrocnemius medialis (GM), gastrocnemius lateralis (GL), semitendinosus (ST), and biceps femoris (BF) muscles.*

| **(a)** | | |
| --- | --- | --- |
| **Muscle** | Coefficient of concordance in OA subjects | **Standard deviation** |
| **RF** | 0.65 | 0.19 |
| **VL** | 0.77 | 0.11 |
| **VM** | 0.73 | 0.13 |
| **TA** | 0.59 | 0.14 |
| **GM** | 0.56 | 0.19 |
| **GL** | 0.58 | 0.19 |
| **ST** | 0.68 | 0.19 |
| **BF** | 0.62 | 0.14 |
| **Average** | 0.65 |  |
| **(b)** | | |
| **Muscle** | Coefficient of concordance in healthy subjects | **Standard deviation** |
| **RF** | 0.60 | 0.07 |
| **VL** | 0.75 | 0.10 |
| **VM** | 0.72 | 0.10 |
| **TA** | 0.65 | 0.14 |
| **GM** | 0.54 | 0.16 |
| **GL** | 0.44 | 0.14 |
| **ST** | 0.78 | 0.15 |
| **BF** | 0.59 | 0.15 |
| **Average** | 0.63 |  |

**References**

1 Giroux, M., Moissenet, F. & Dumas, R. EMG-based validation of musculo-skeletal models for gait analysis. *Computer methods in biomechanics and biomedical engineering* **16**, 152-154 (2013).

2 Moissenet, F., Chèze, L. & Dumas, R. A 3D lower limb musculoskeletal model for simultaneous estimation of musculo-tendon, joint contact, ligament and bone forces during gait. *Journal of Biomechanics* **47**, 50-58 (2014).
