# Supplementary material for: Knee loading in OA subjects is correlated to flexion and adduction moments and to contact point locations
Source: Sci Rep. 2021 Apr 21;11:8594. doi: 10.1038/s41598-021-87978-2 (PMC8060429; doi:10.1038/s41598-021-87978-2)

Knee loading in OA subjects is correlated to flexion and adduction moments and to contact point locations

Ali Zeighami^1^, Raphael Dumas^2^, Rachid Aissaoui^1*^

1. Laboratoire de Recherche en Imagerie et Orthopédie (LIO), École de Technologie Supérieure (ÉTS), Centre de Recherche du CHUM, Montréal, Québec, Canada
2. Univ Lyon, Univ Gustave Eiffel, LBMC UMR_T9406, F69622, Lyon, France

**Supplementary material 3:**

**Knee adduction and flexion moments during the stance phase**

**Knee adduction and flexion moments during the stance phase**

The knee adduction moments (KAM) and knee flexion moments (KFM) of the 10 healthy (H01 – H10) and 12 OA (OA01 – OA12) subjects are presented in the following plots. The KAM and KFM are averaged from all the gait cycles performed during 45 seconds of treadmill walking. Only the stance phase is analyzed. The dashed lines represent the standard deviations (±1 SD) around the average. The KAM and KFM are normalized to body weight * height (BW*height) and to 100% of the gait stance. The figures on the left show all the individual curves at each cycle superimposed.


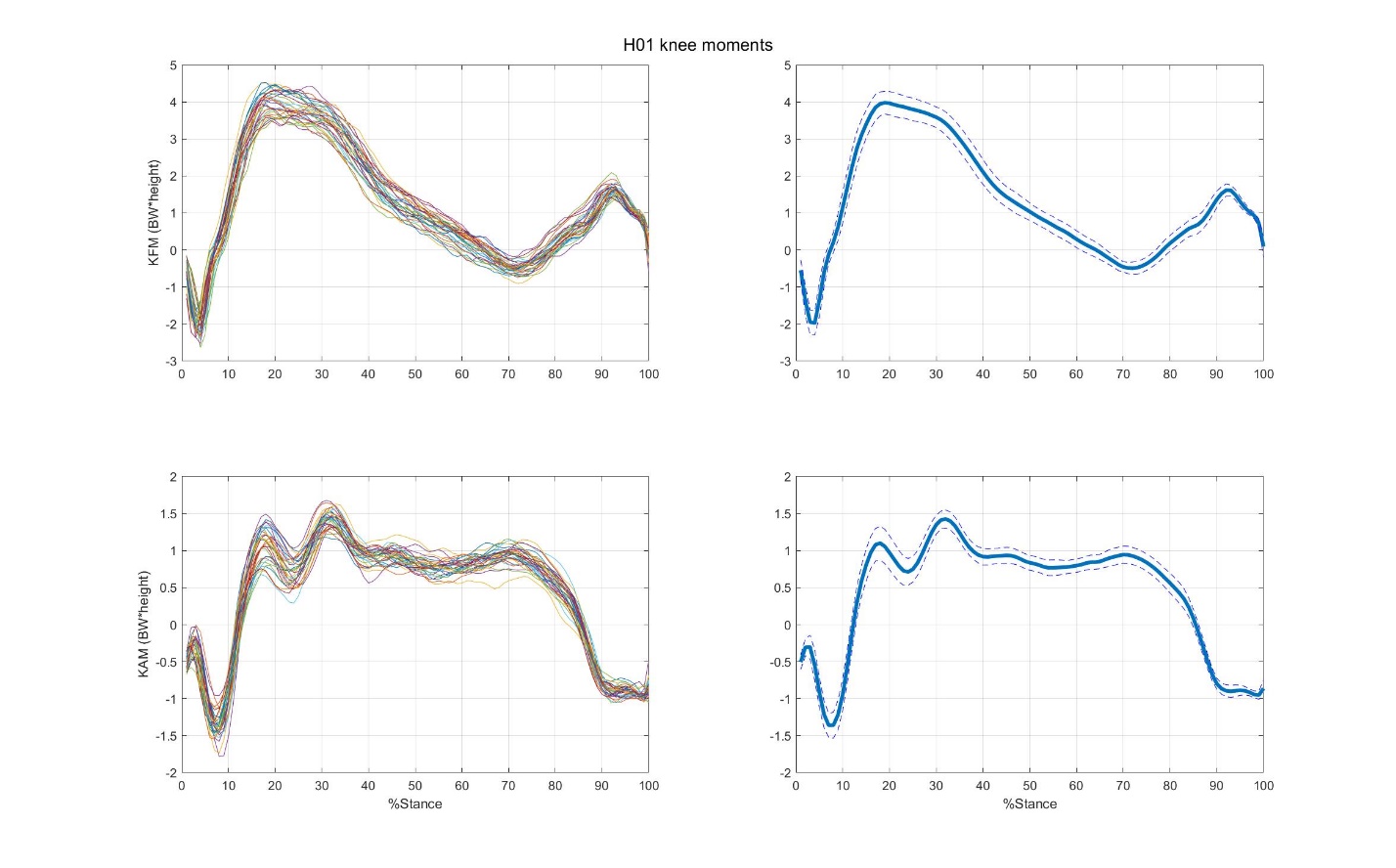

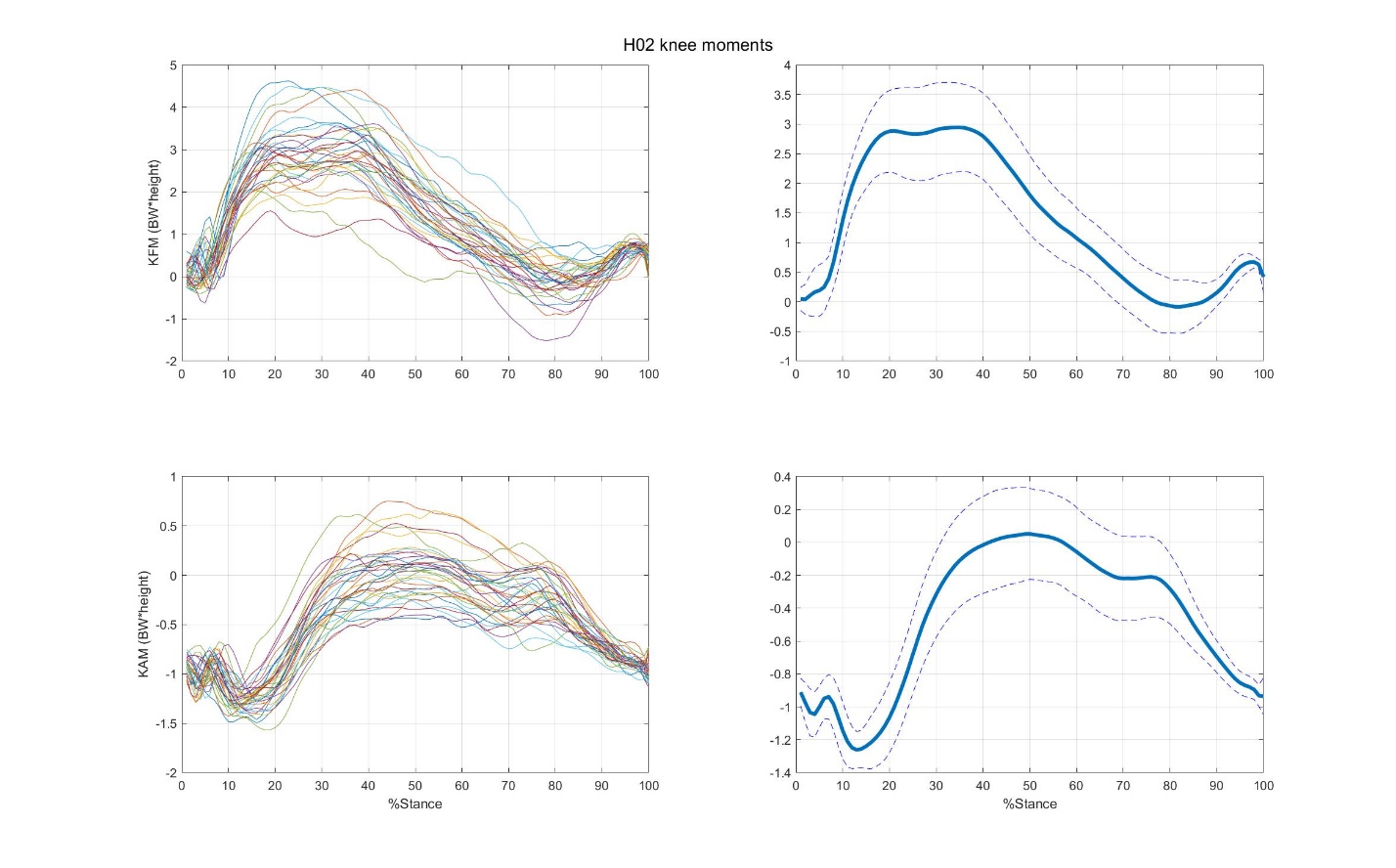

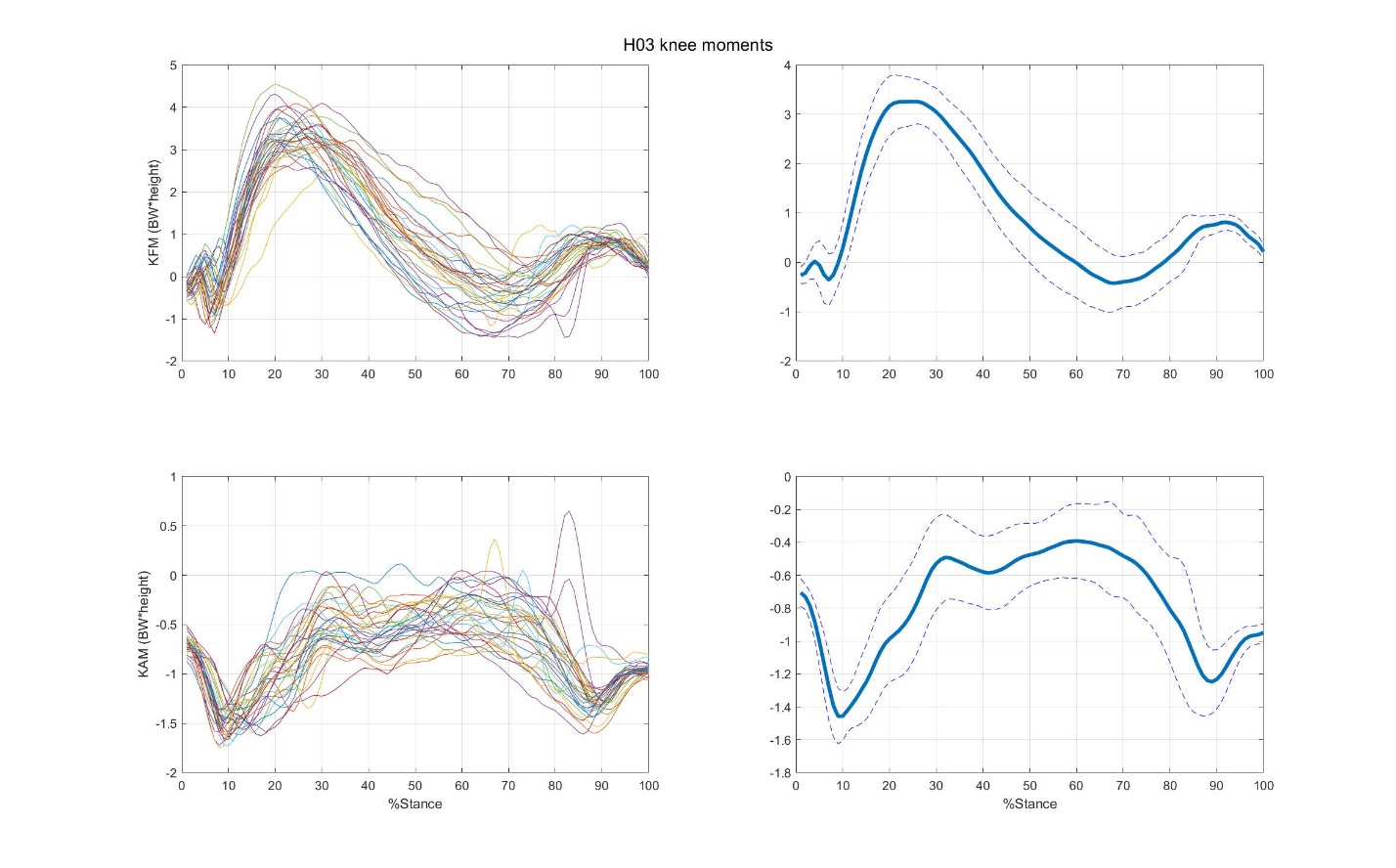

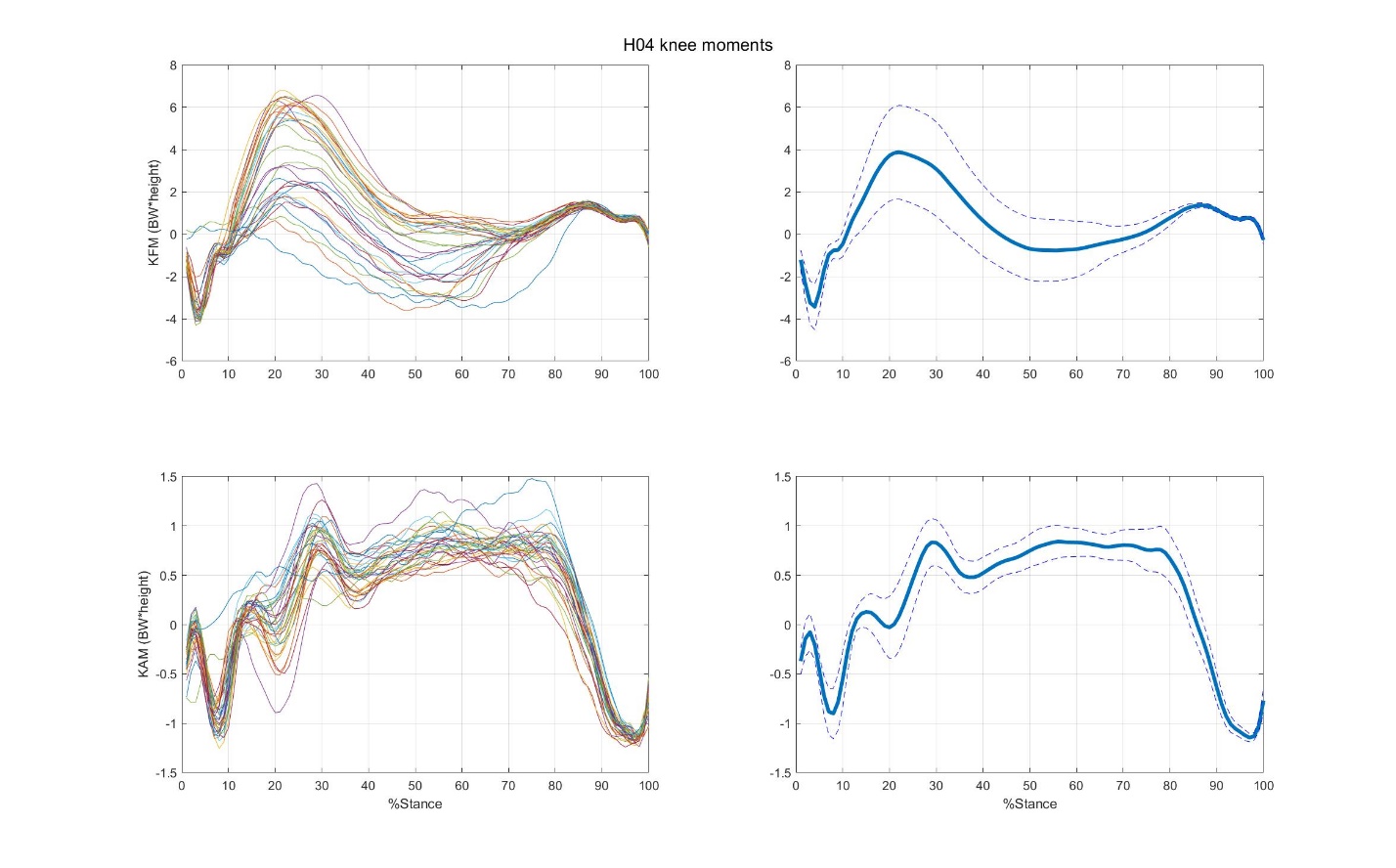

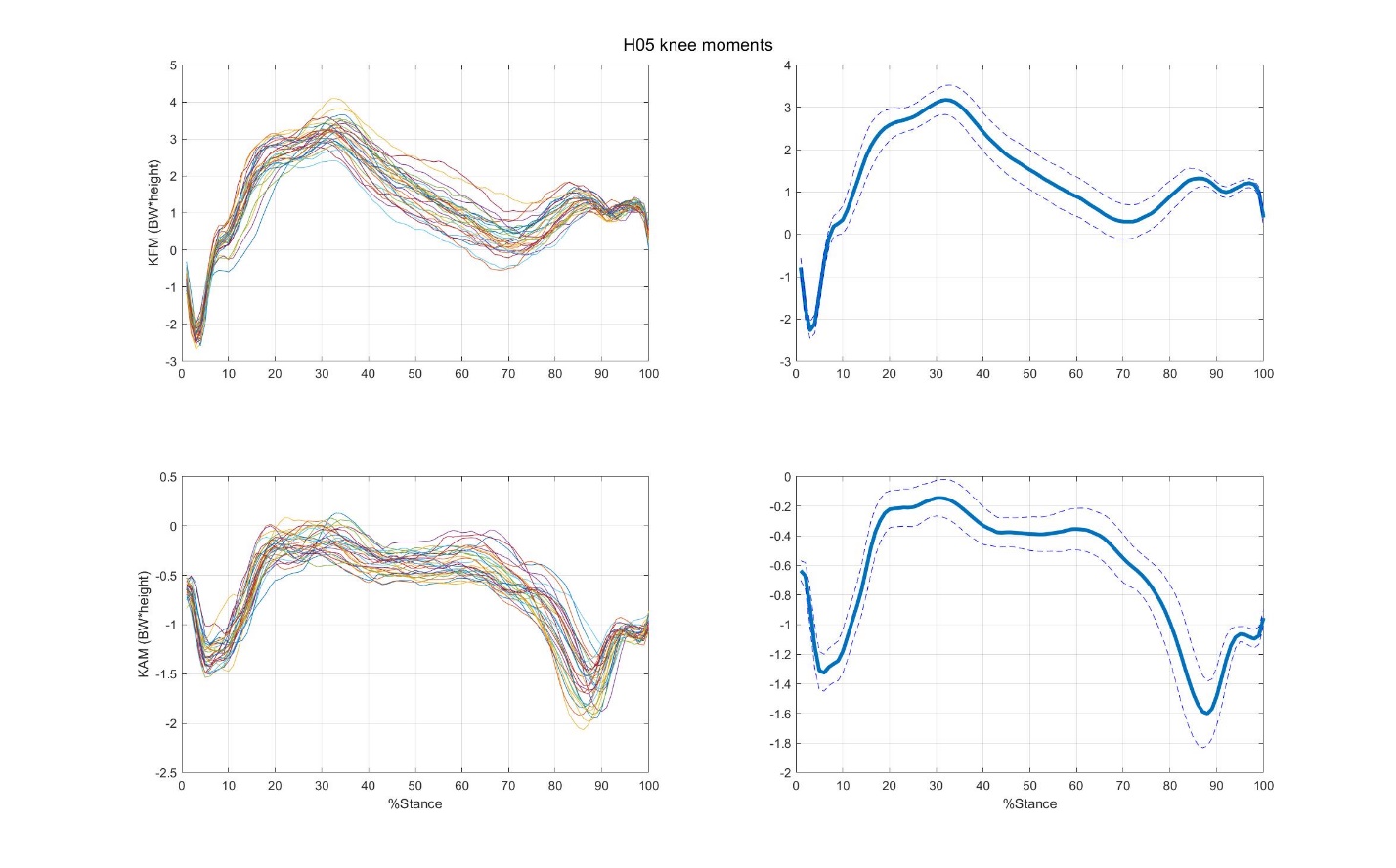

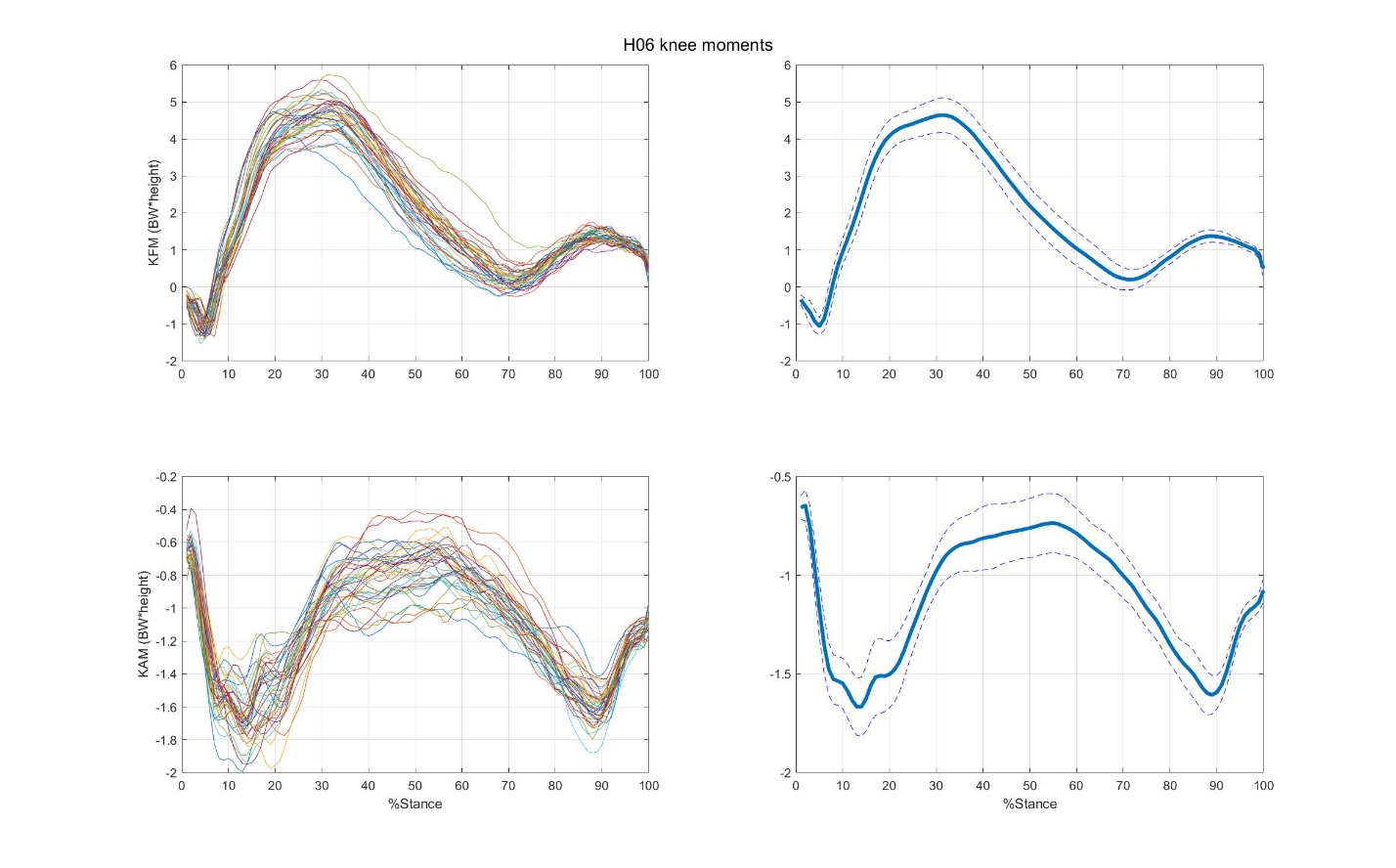

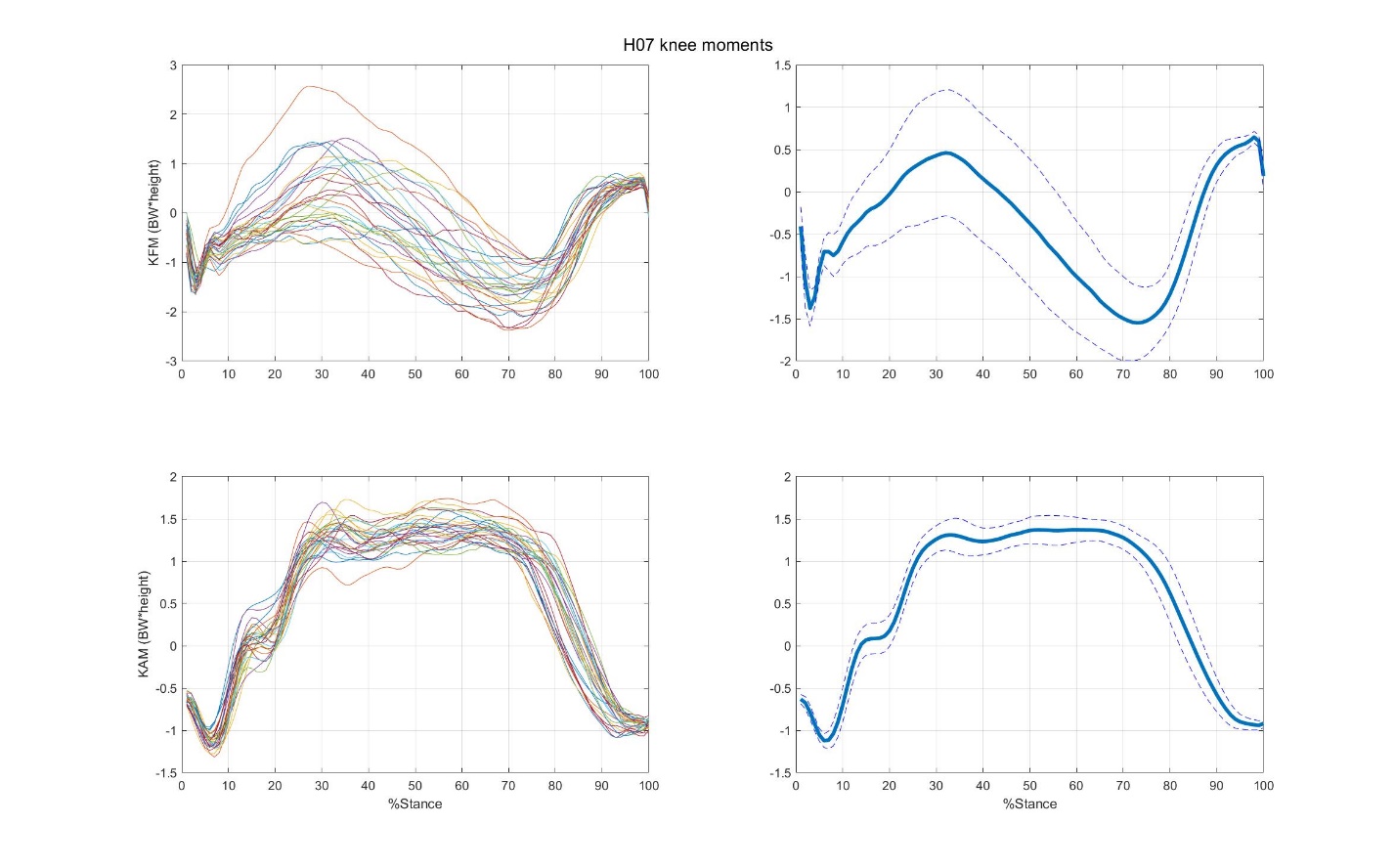

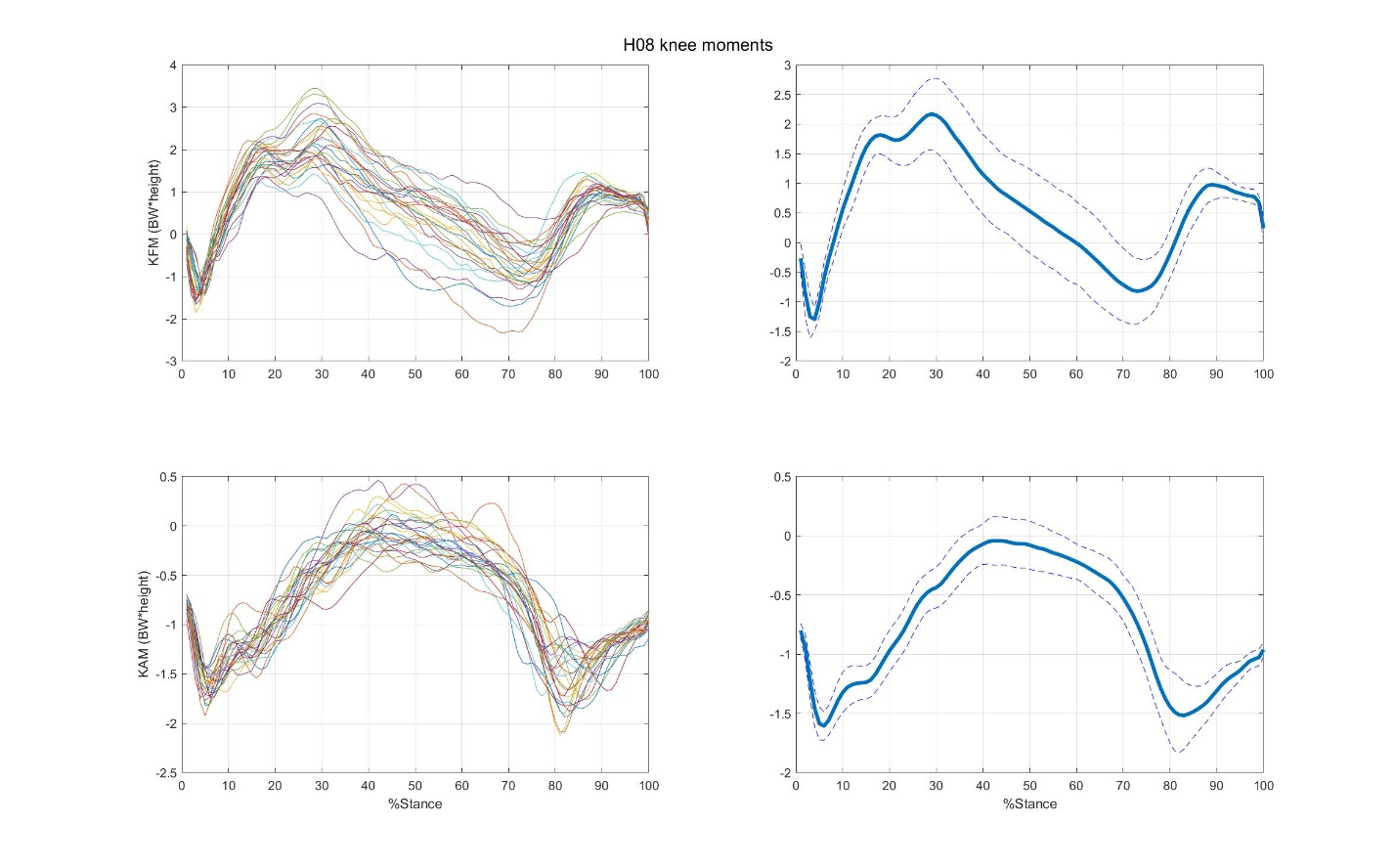

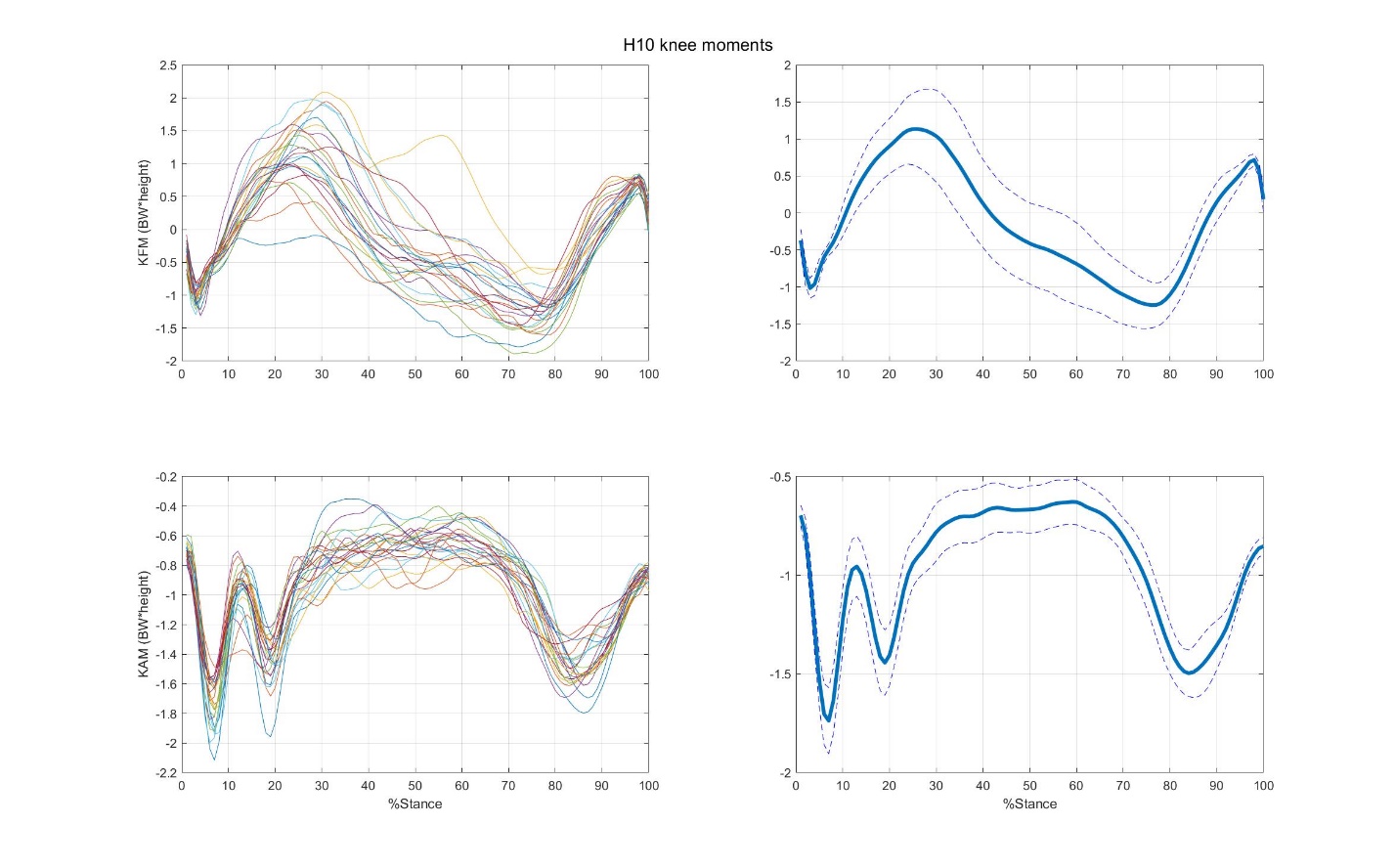

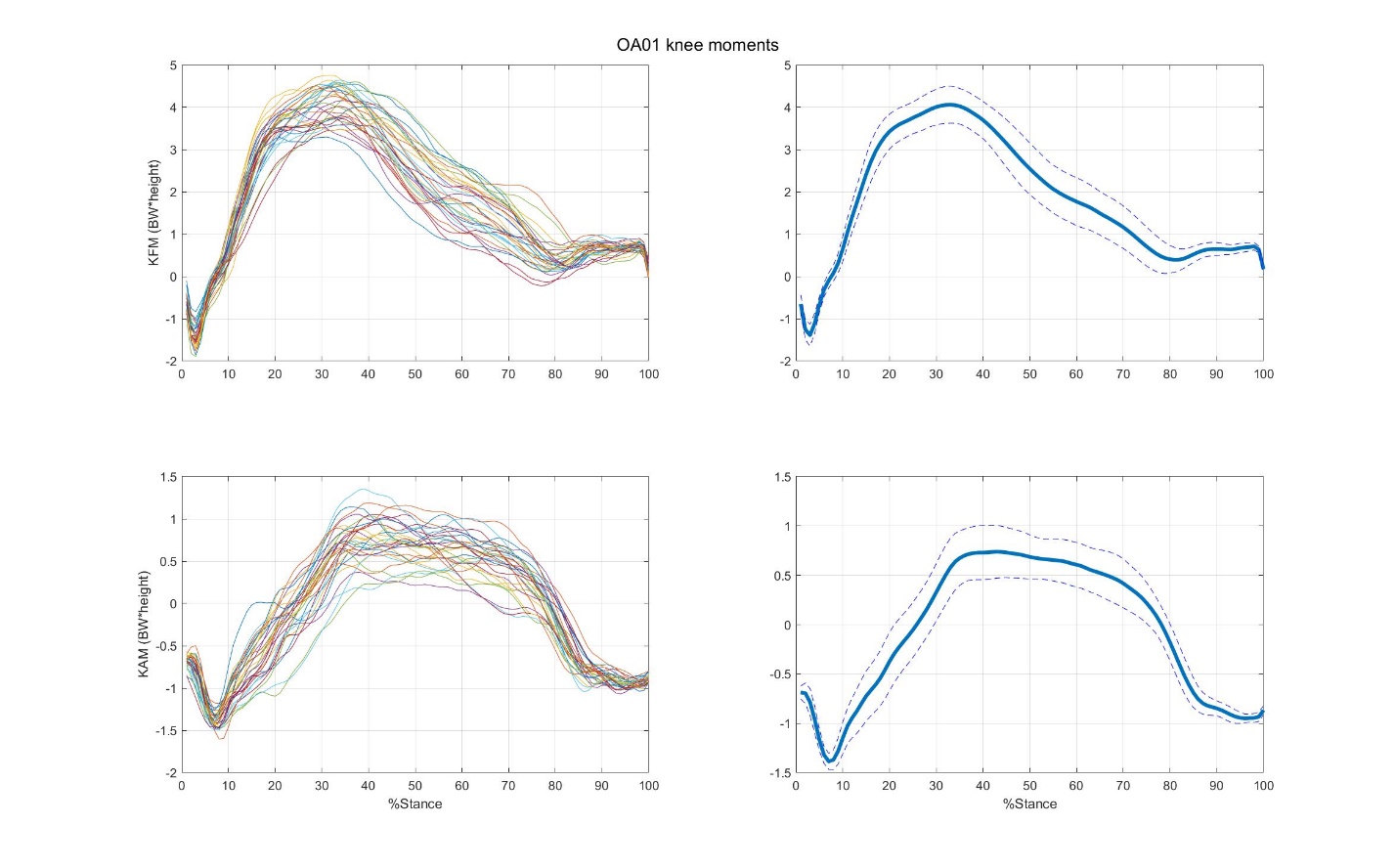

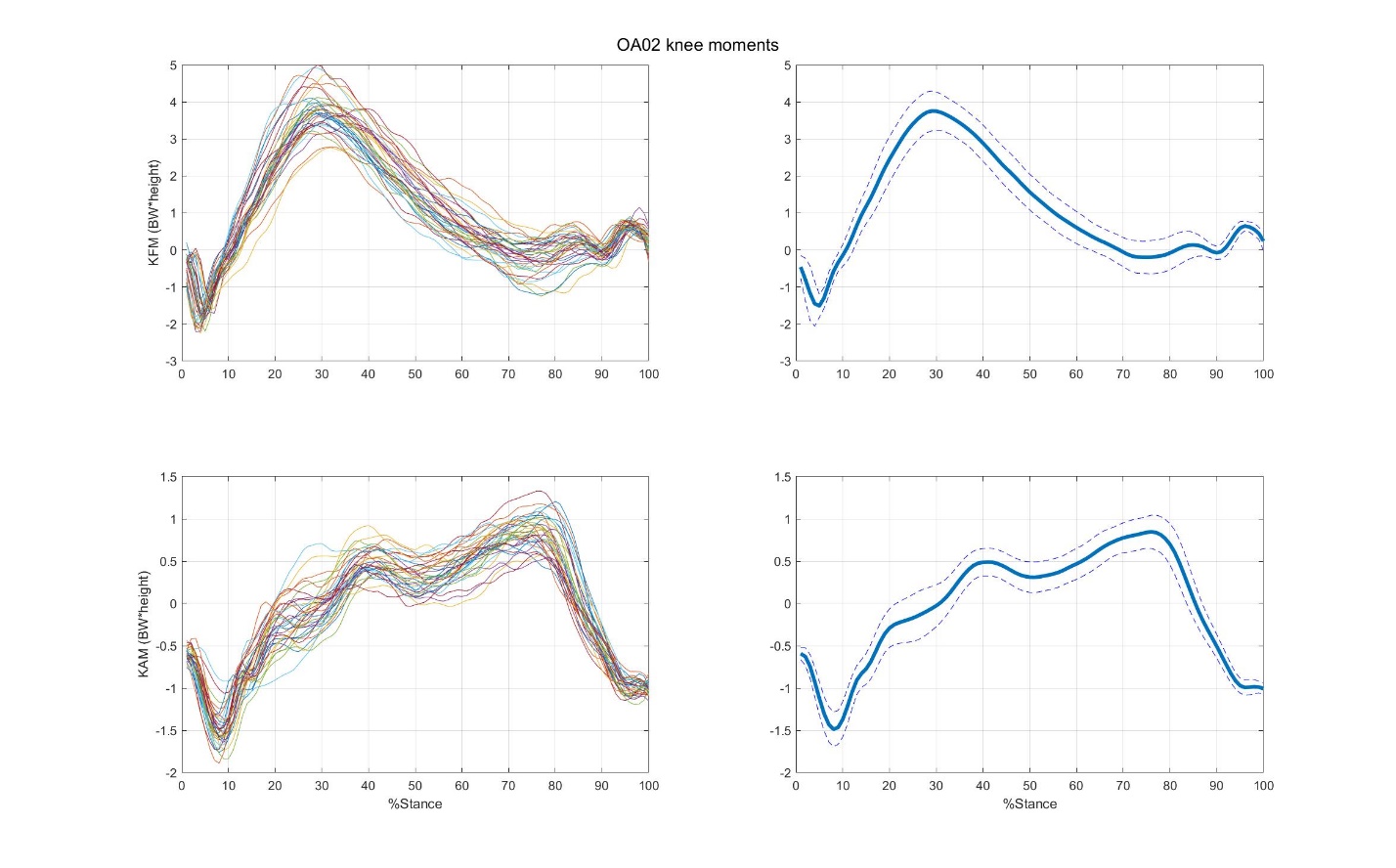

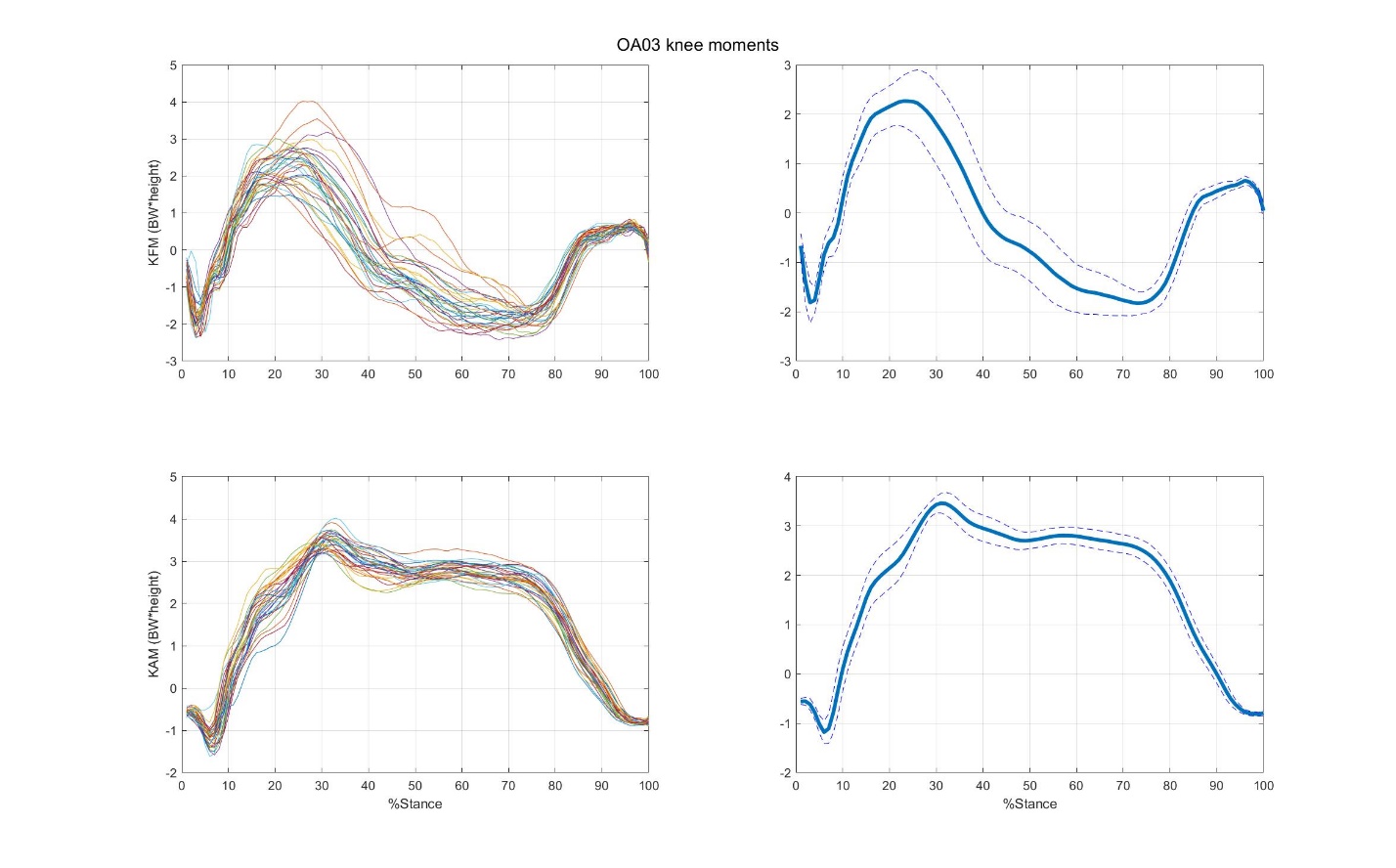

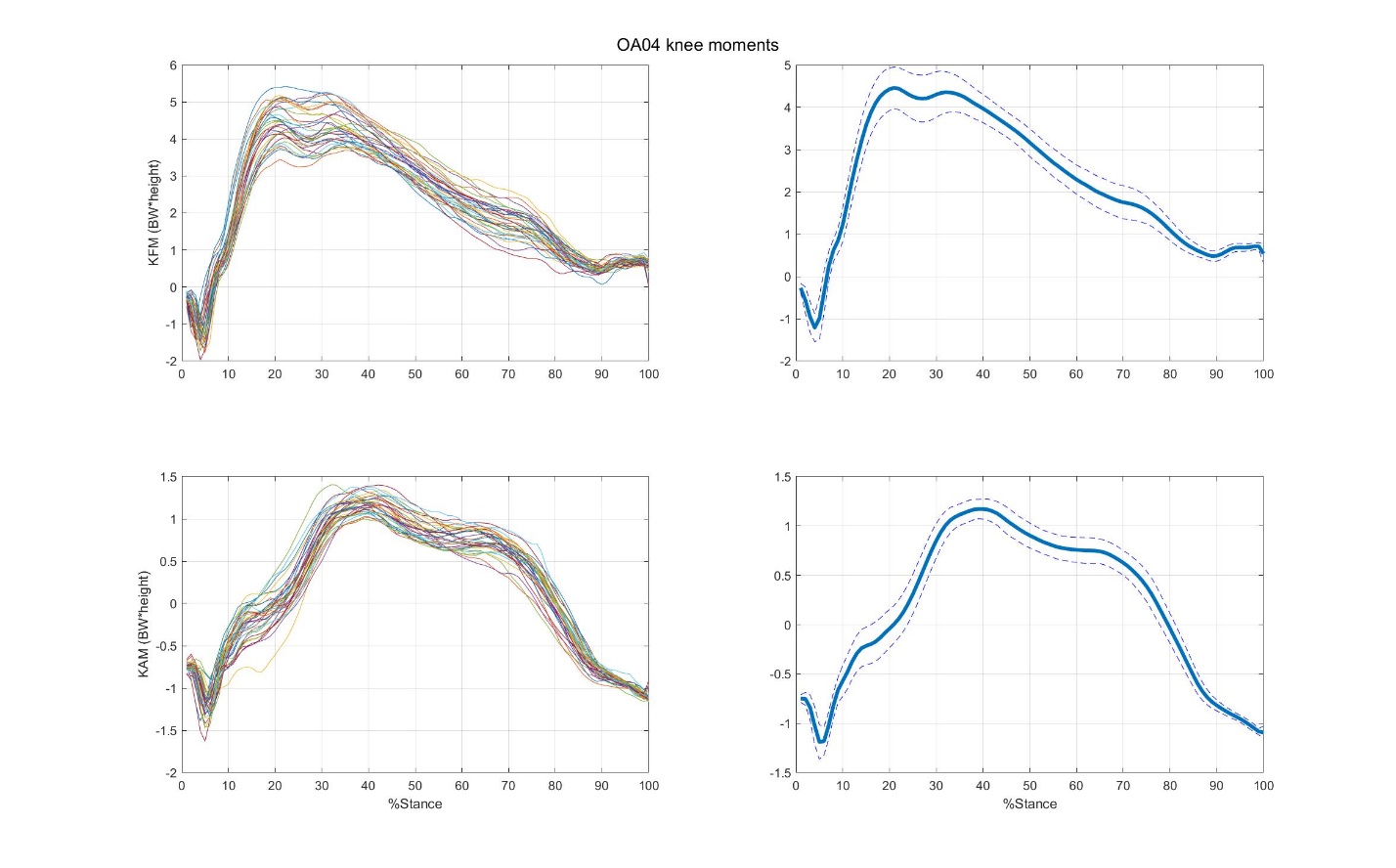

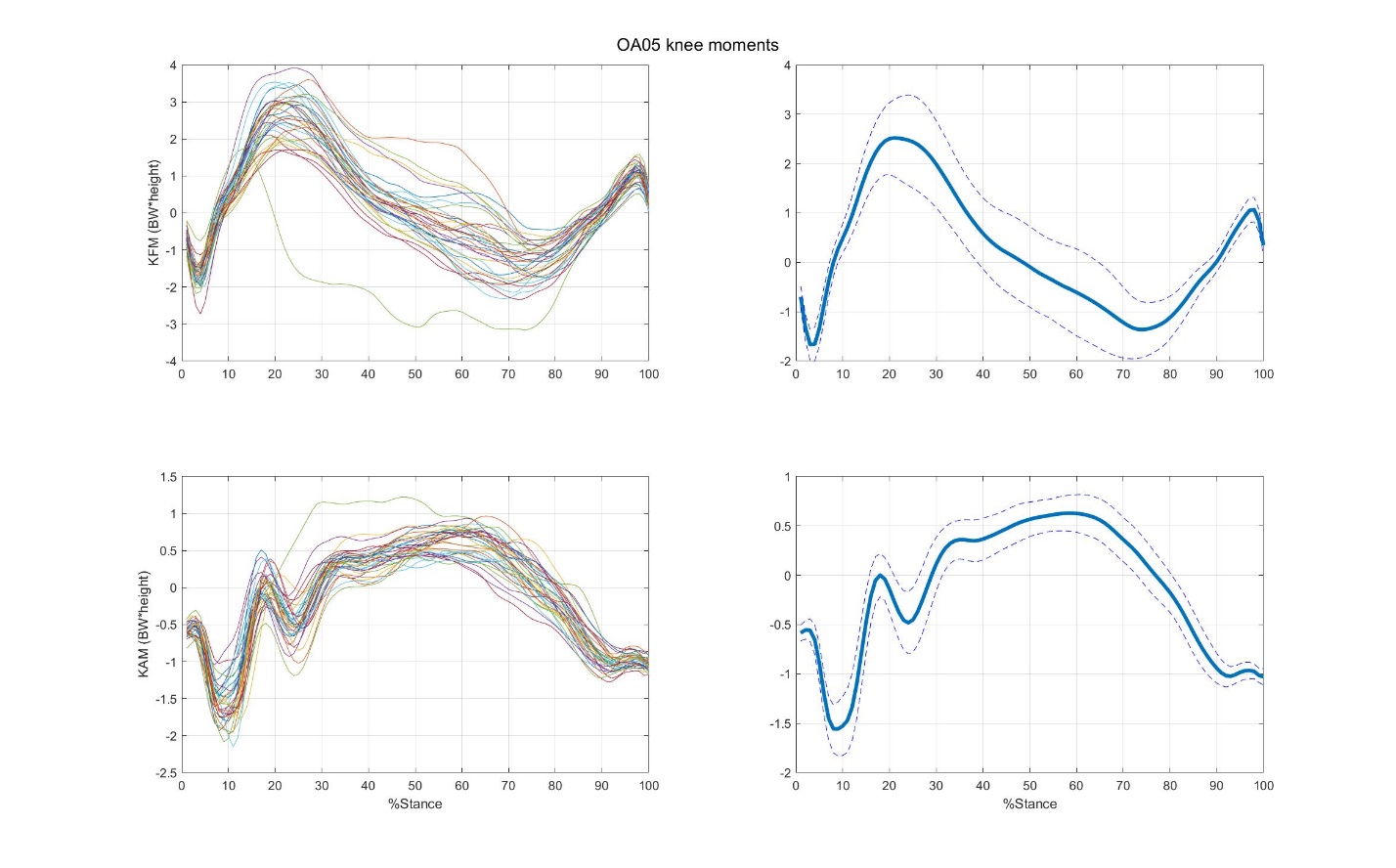

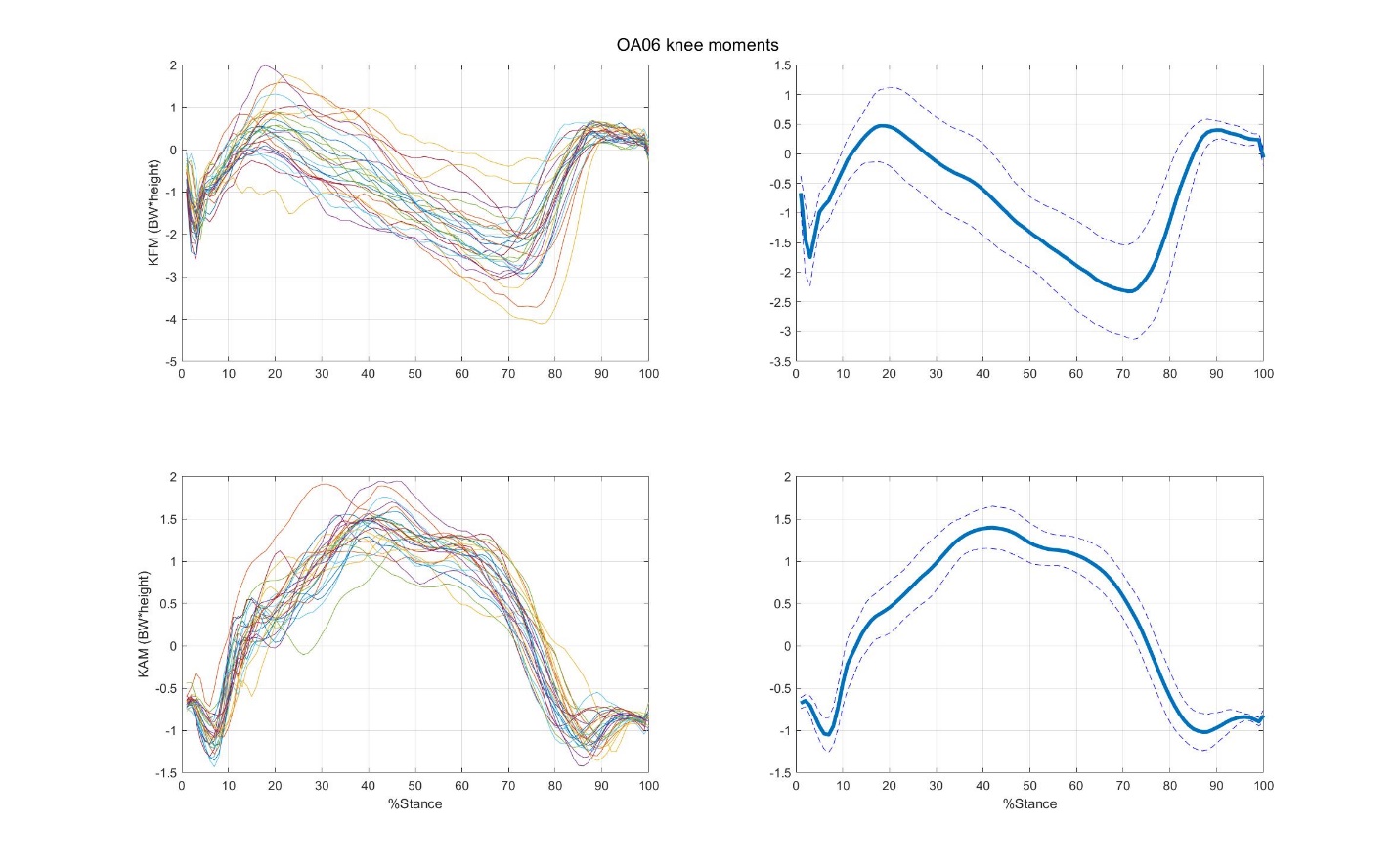

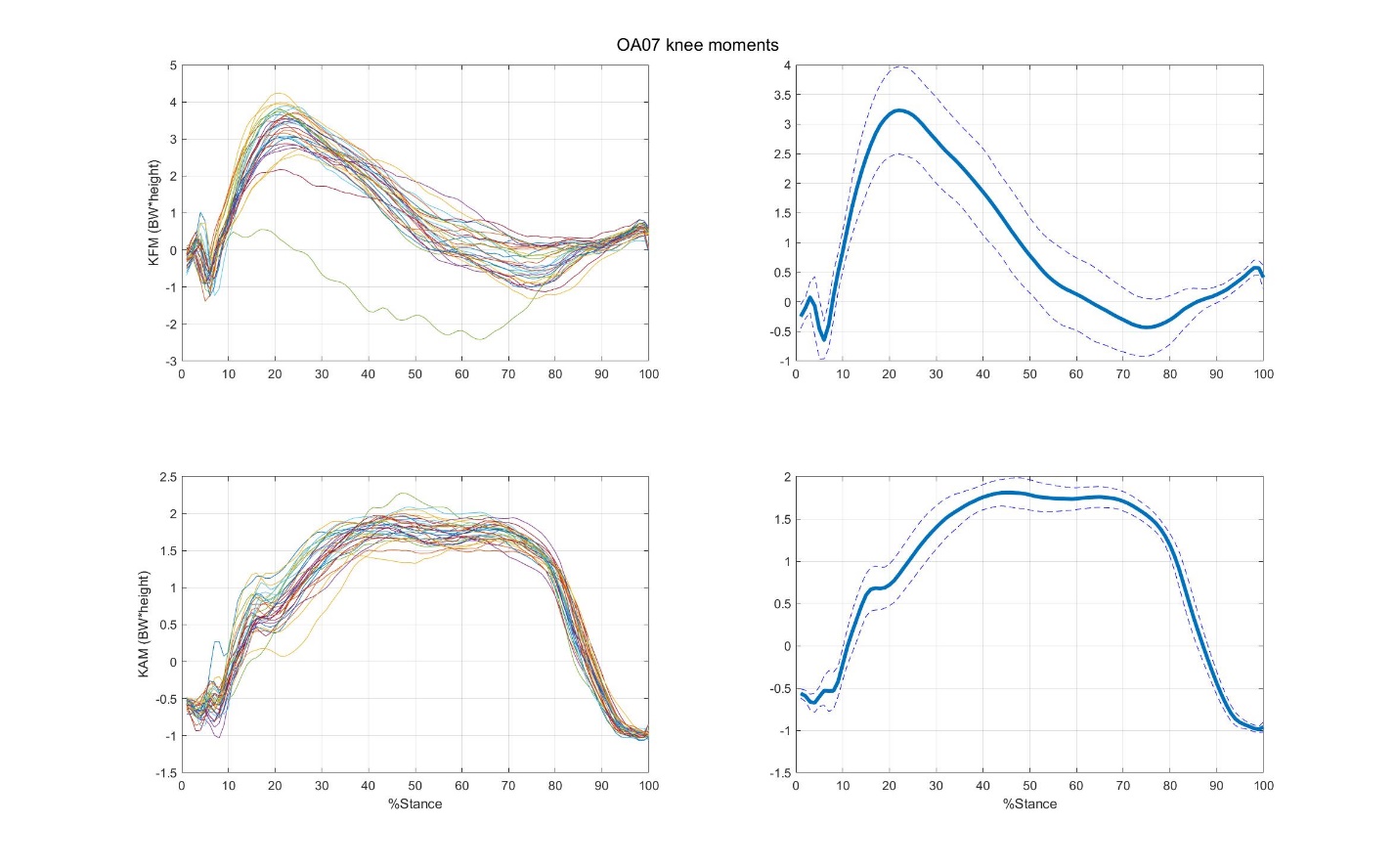

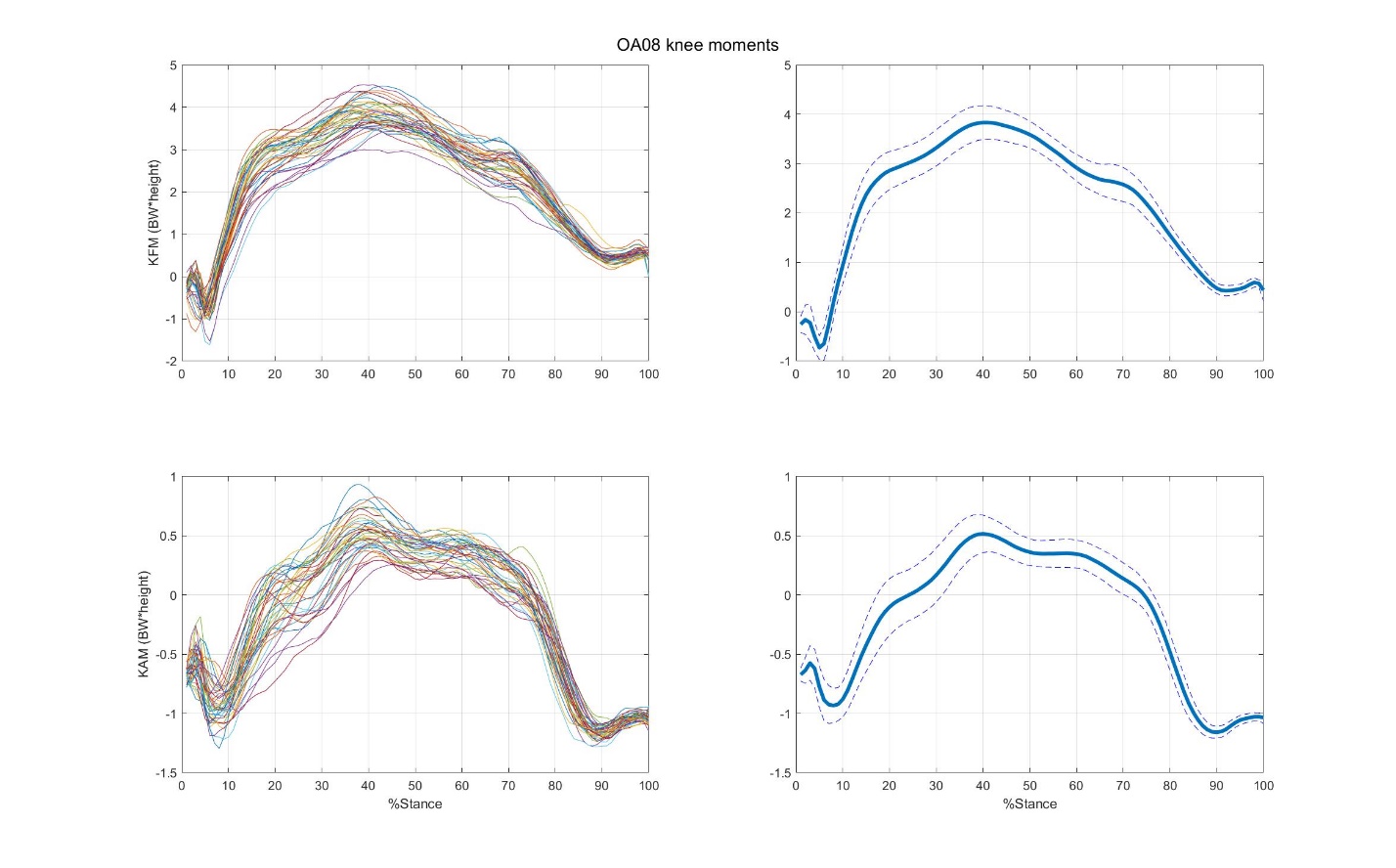

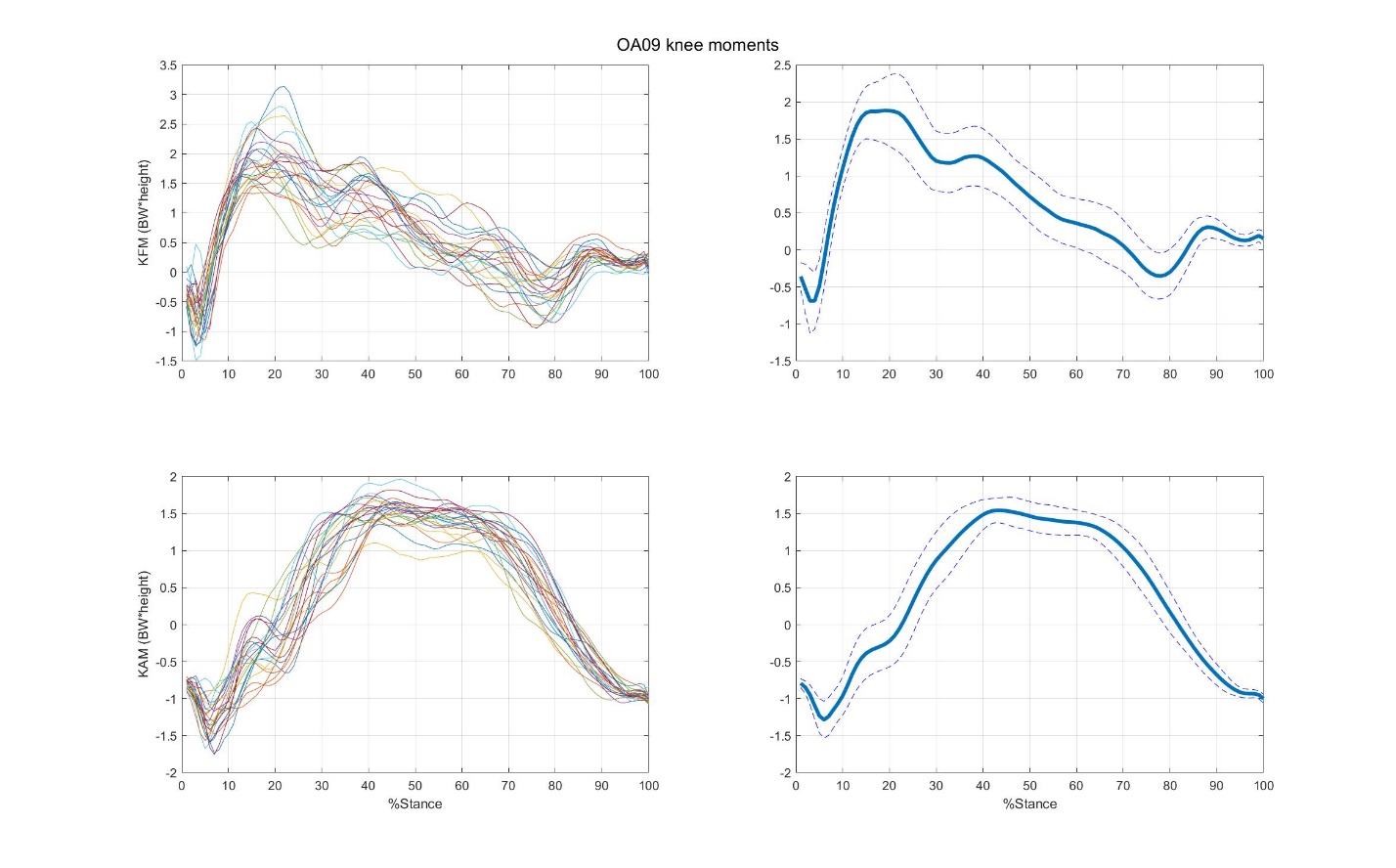

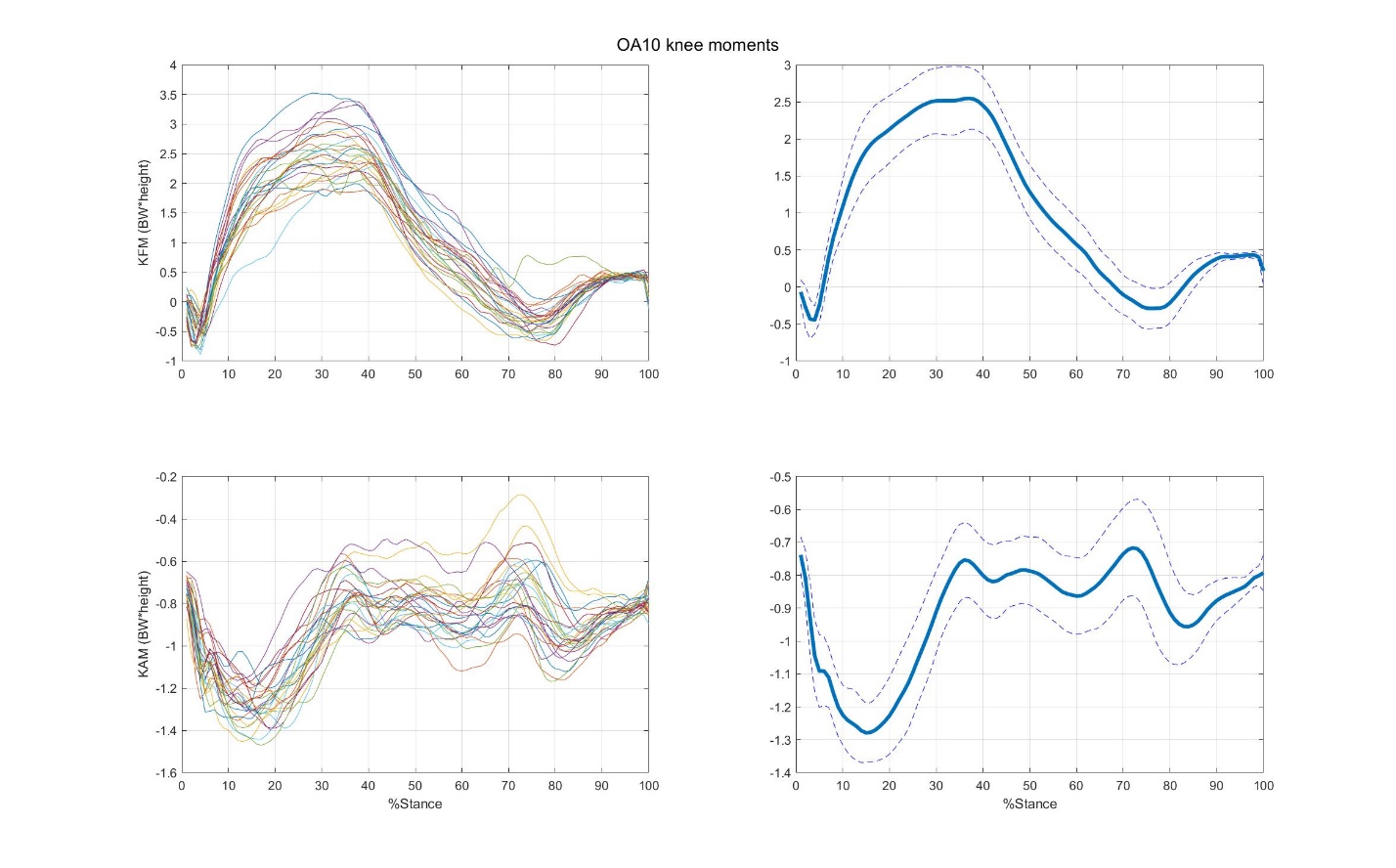

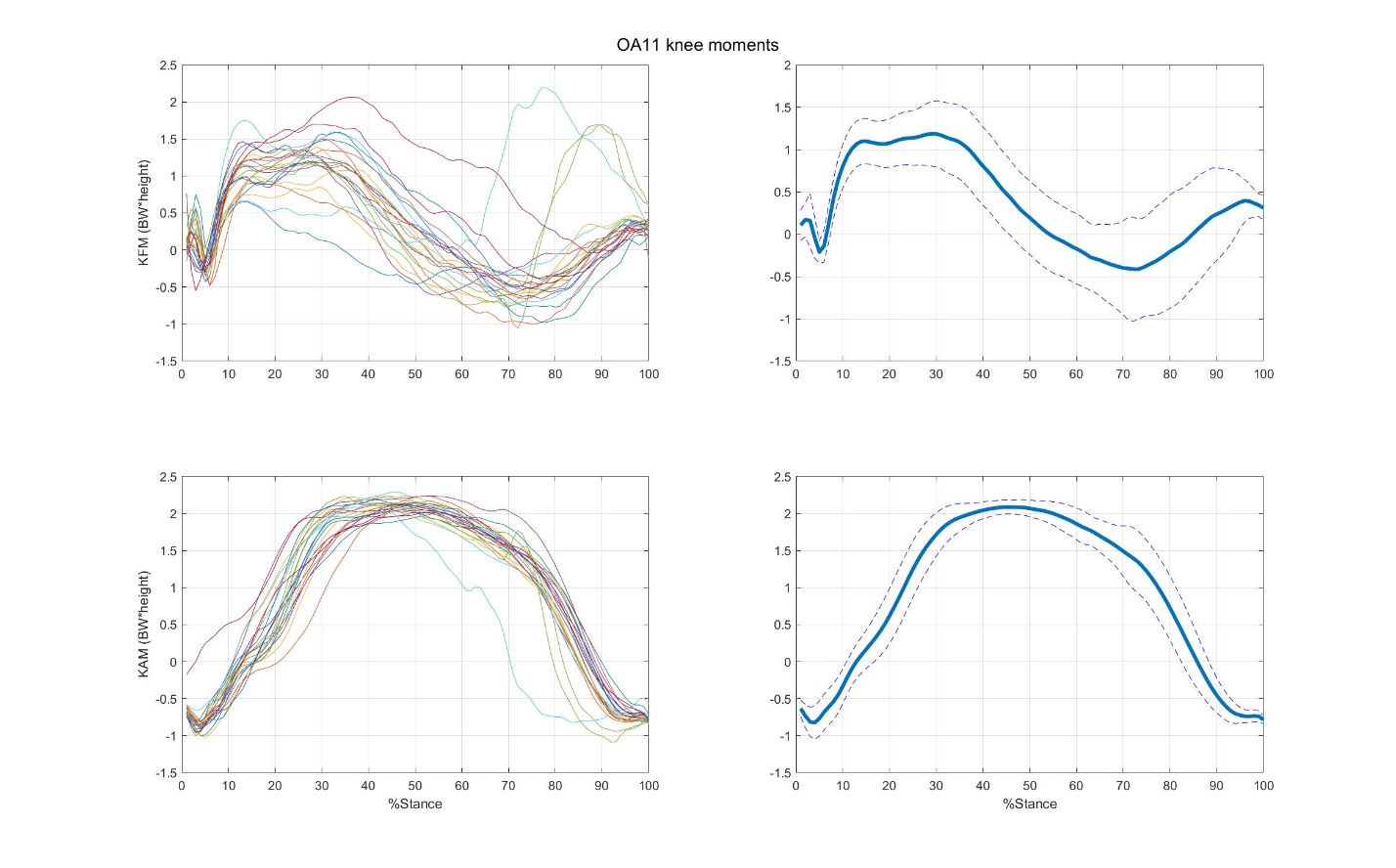

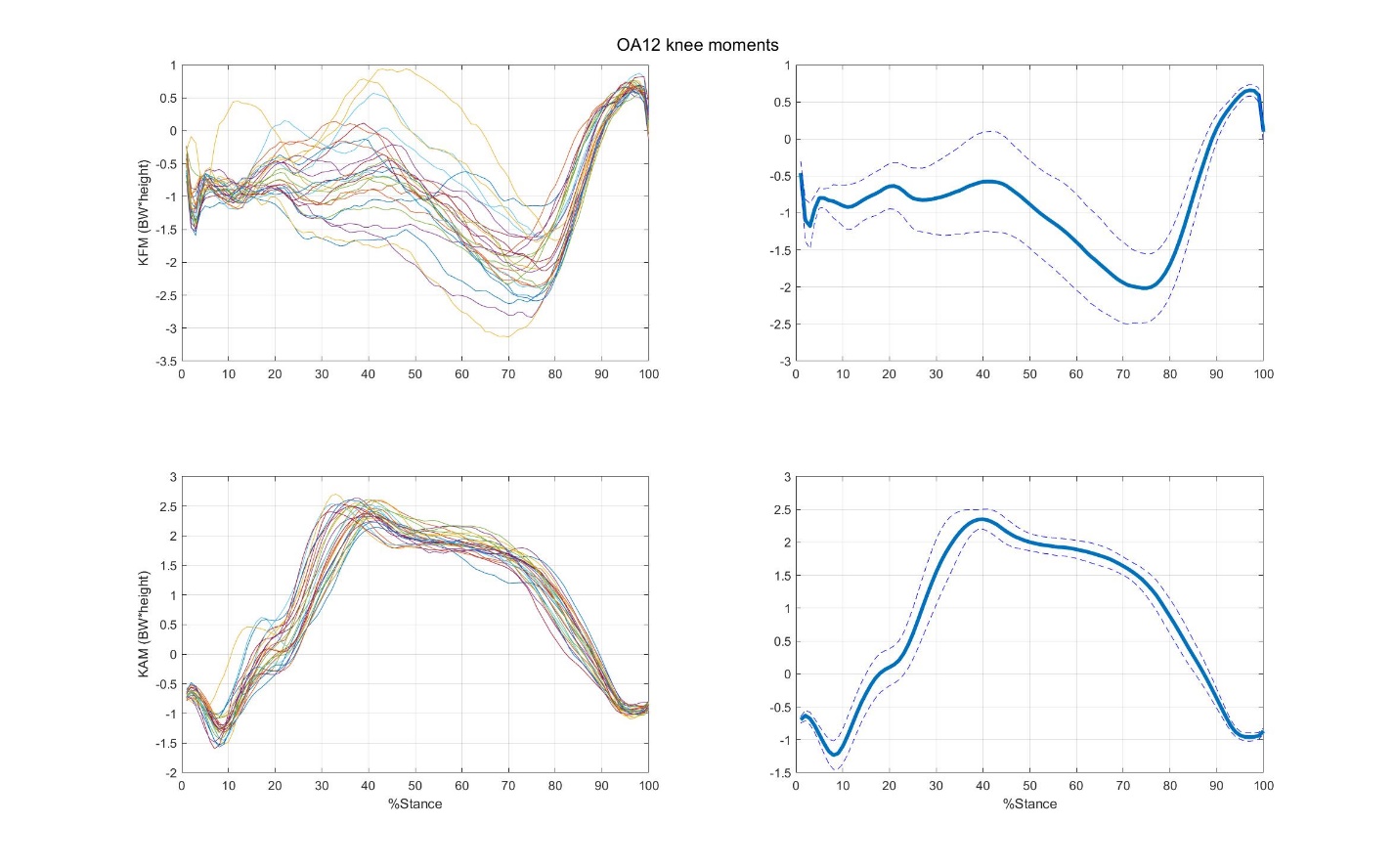

Supplement: Supplementary file 3 — Supplementary Information 3. [file 41598_2021_87978_MOESM3_ESM.docx]
