# Supplementary material for: Knee loading in OA subjects is correlated to flexion and adduction moments and to contact point locations
Source: Sci Rep. 2021 Apr 21;11:8594. doi: 10.1038/s41598-021-87978-2 (PMC8060429; doi:10.1038/s41598-021-87978-2)

Knee loading in OA subjects is correlated to flexion and adduction moments and to contact point locations

Ali Zeighami^1^, Raphael Dumas^2^, Rachid Aissaoui^1*^

1. Laboratoire de Recherche en Imagerie et Orthopédie (LIO), École de Technologie Supérieure (ÉTS), Centre de Recherche du CHUM, Montréal, Québec, Canada
2. Univ Lyon, Univ Gustave Eiffel, LBMC UMR_T9406, F69622, Lyon, France

**Supplementary material 4:**

**Contact point trajectories of all subjects over the tibial plateau**

**Contact point trajectories of all subjects over the tibial plateau**

The contact point trajectories of the 10 healthy (H01 – H10) and 12 OA (OA01 – OA12) subjects are presented in the following plots. The contact point trajectories are presented on the subject’s tibial plateau. The figures are a top view of a right knee, where the left knee medial-lateral axis is inverted to keep consistent view (medial plateau on the left). The marks on the contact point trajectories represent the contact point location at the corresponding gait events being timing of the 1^st^ and 2^nd^ peaks of medial and lateral contact forces, heel strike, toe off. The minimum (~0°) and maximum (~70°) knee flexions adopted during the quasi-static acquisitions mark the beginning and the end of each trajectory.


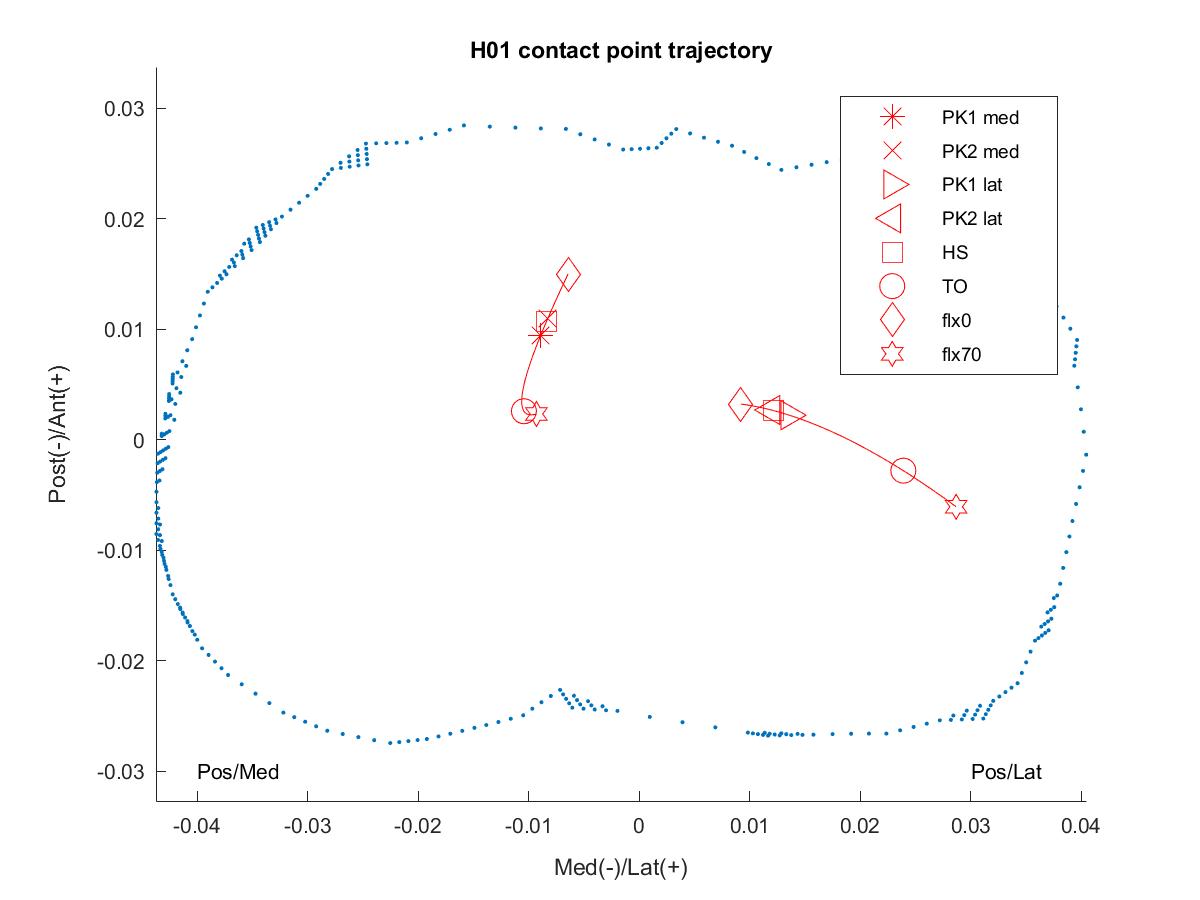

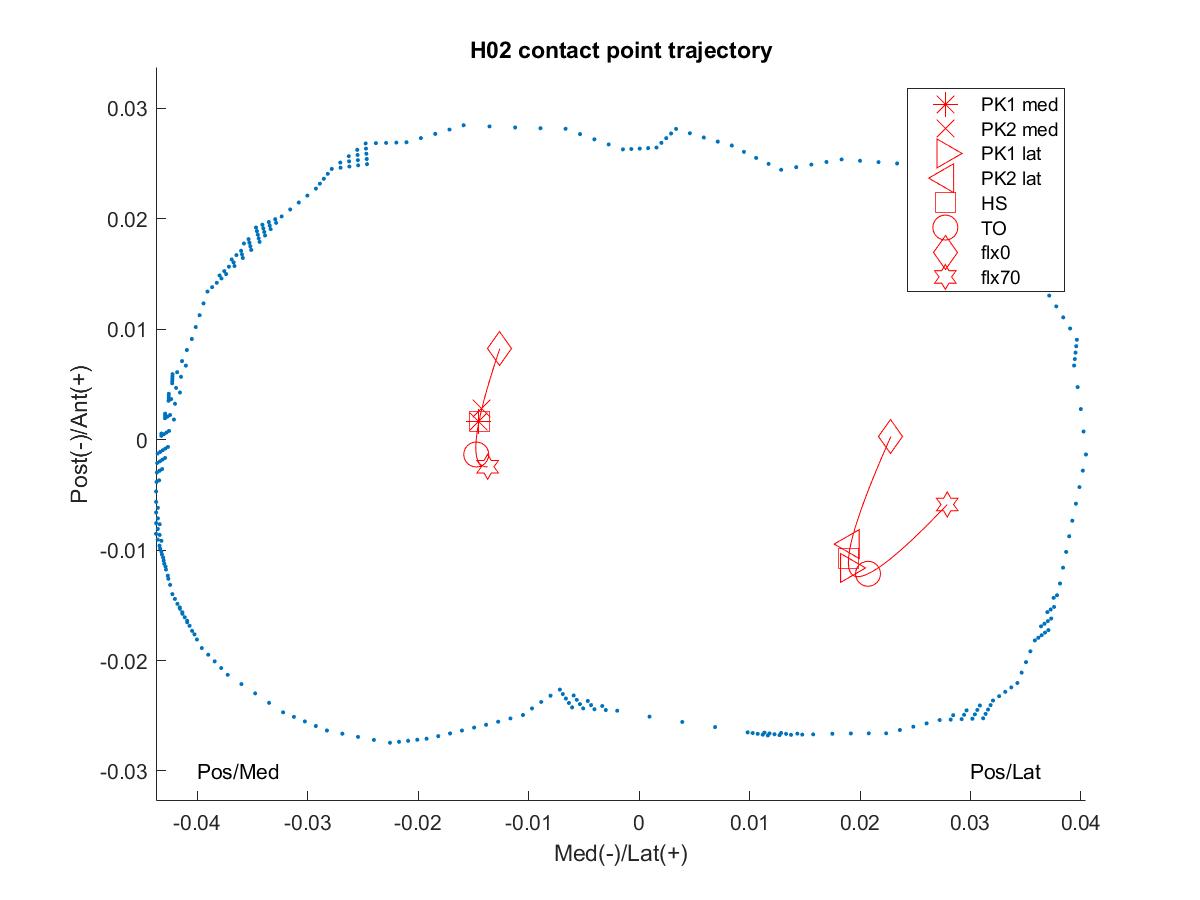

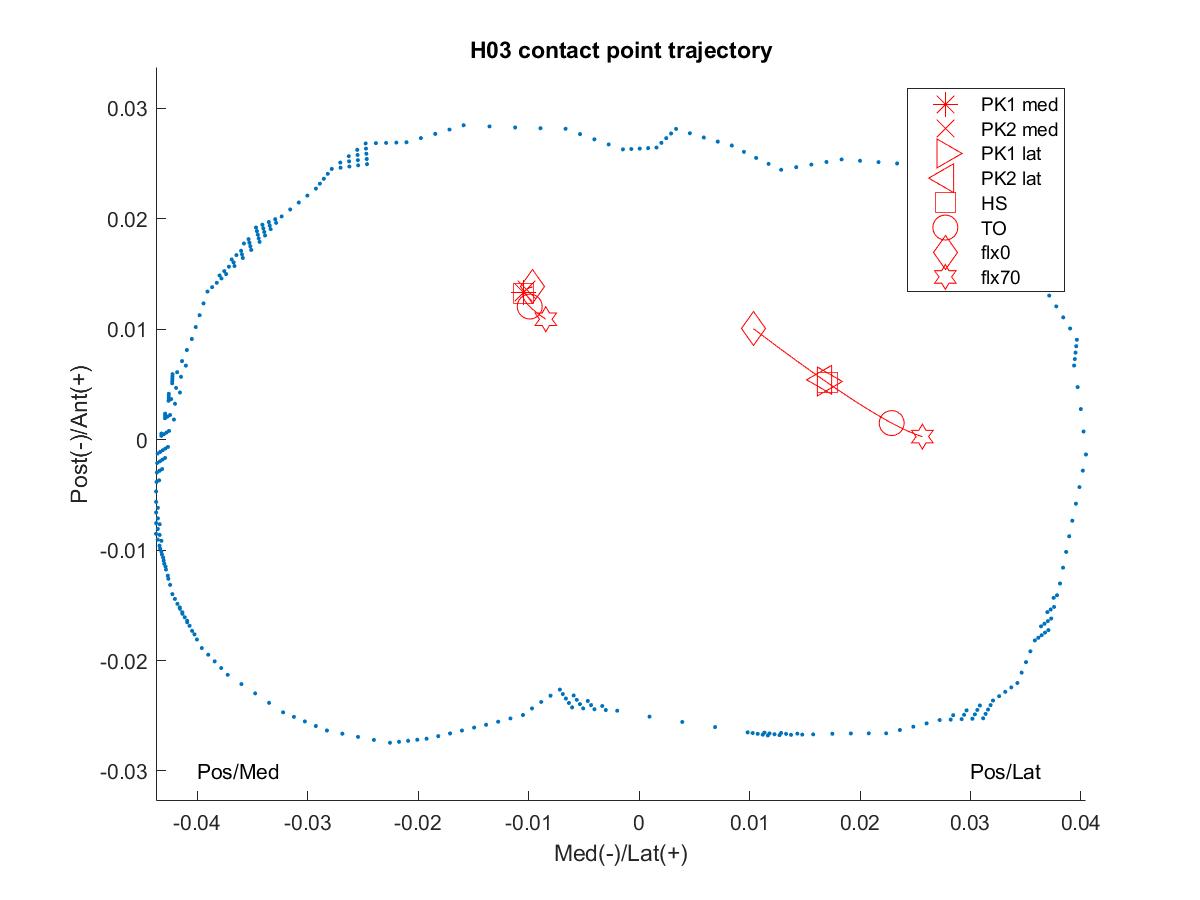

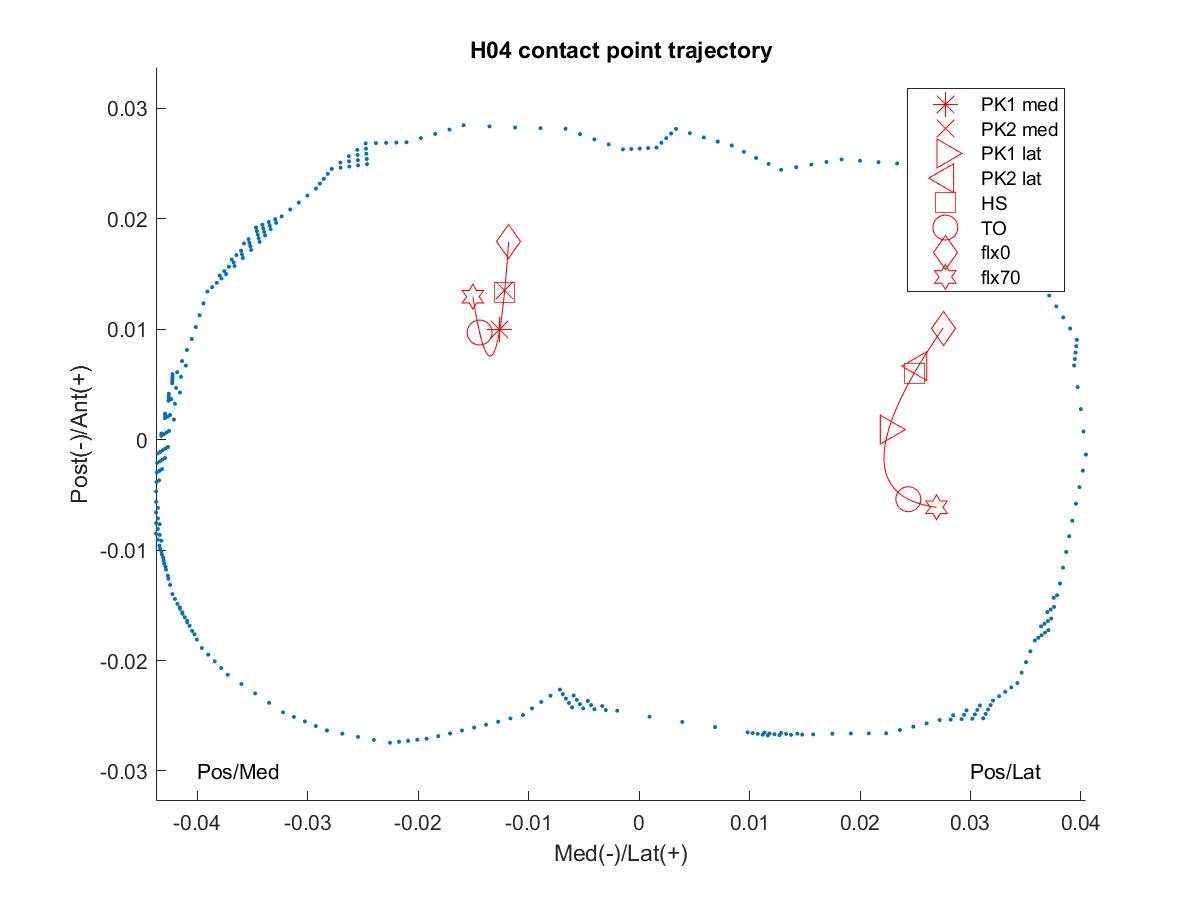

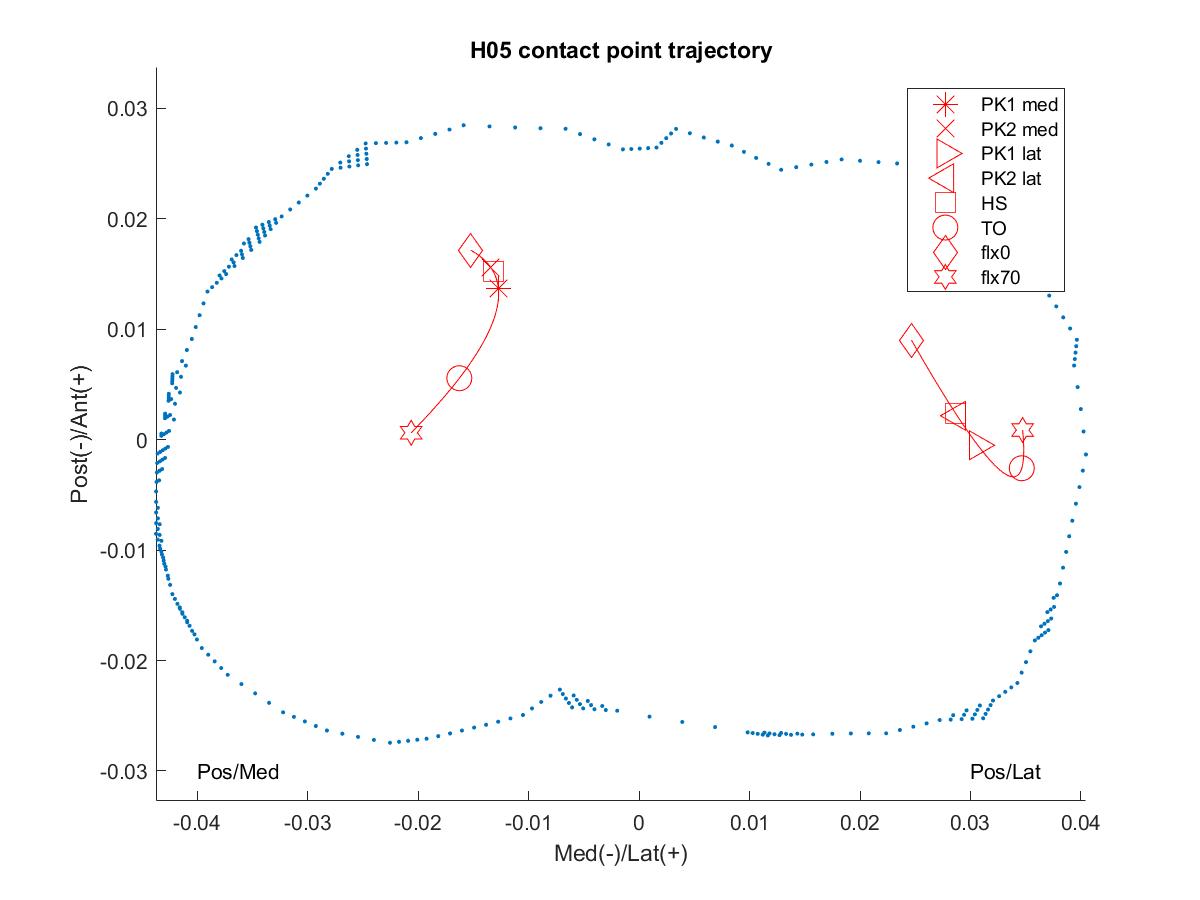

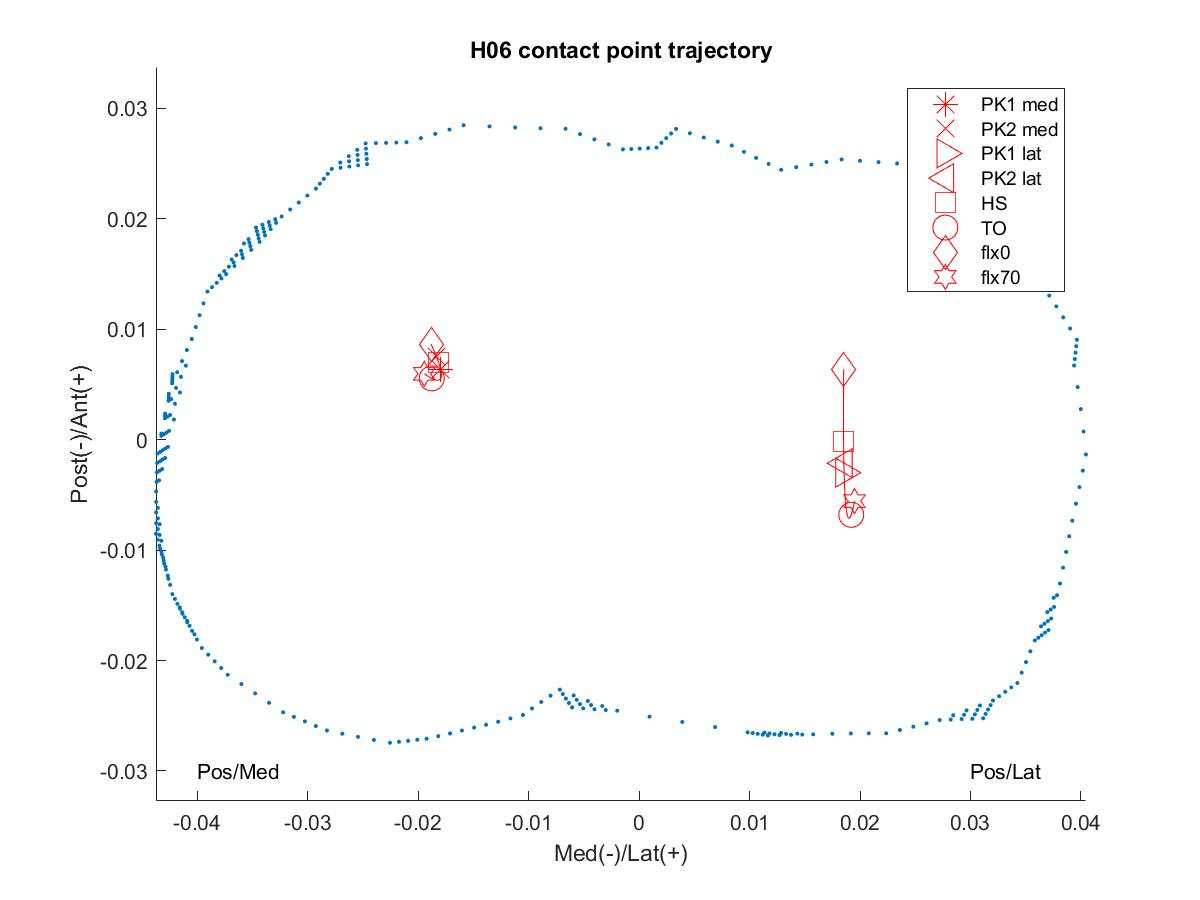

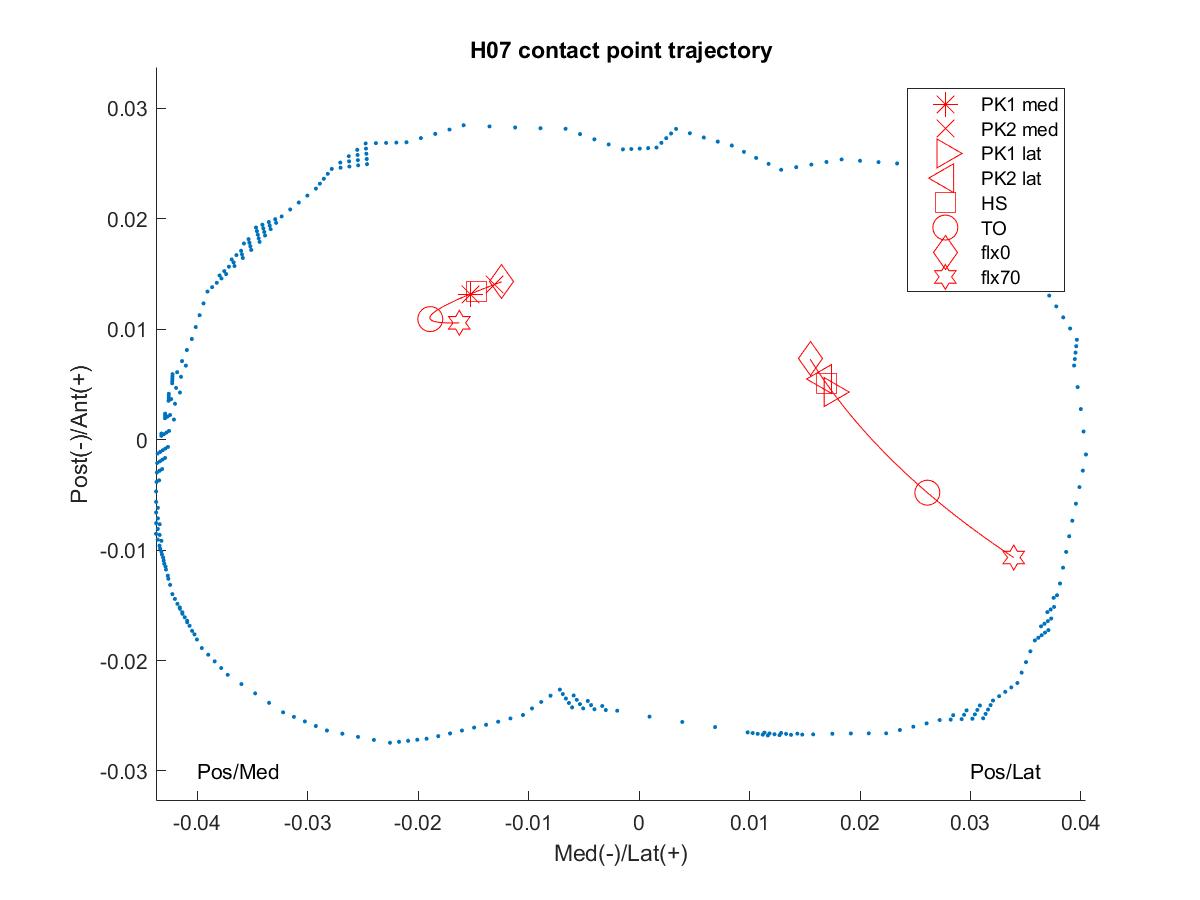

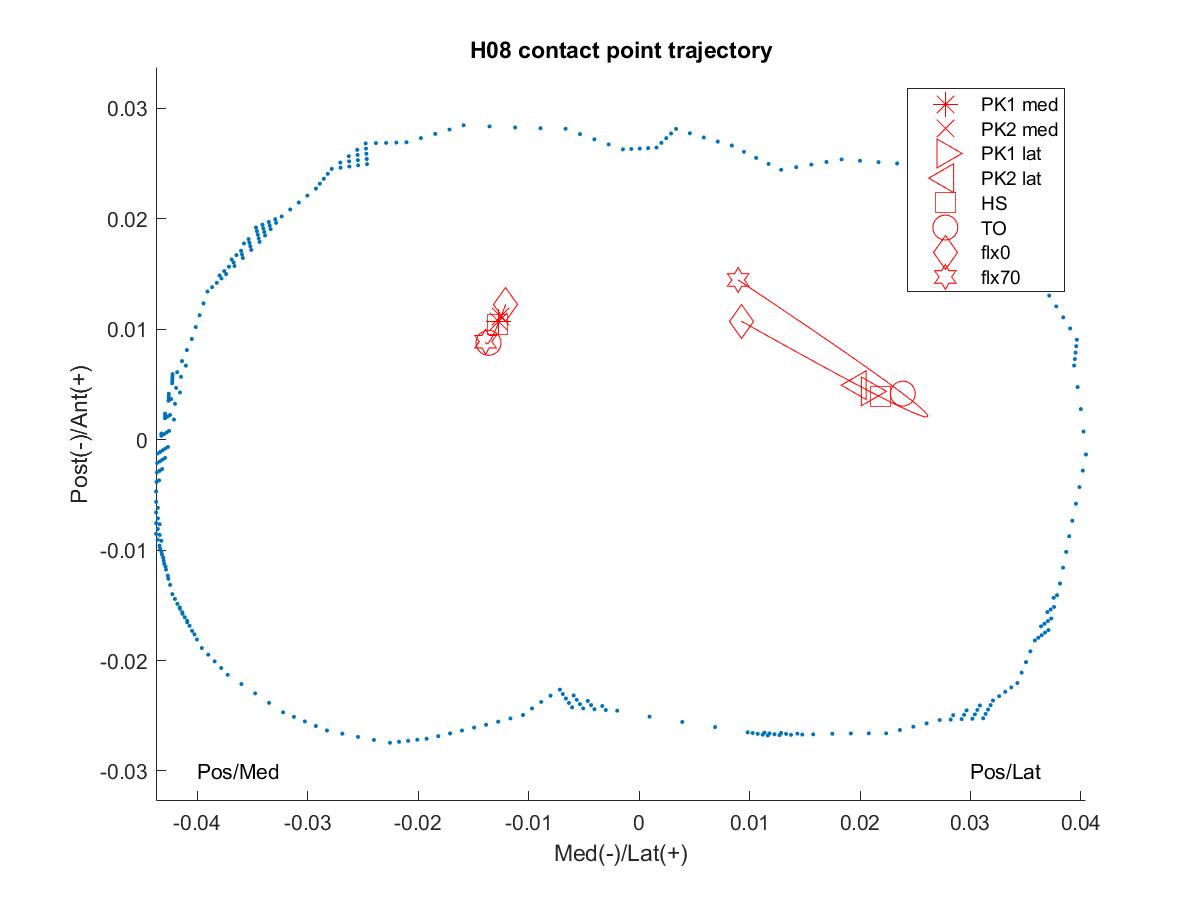

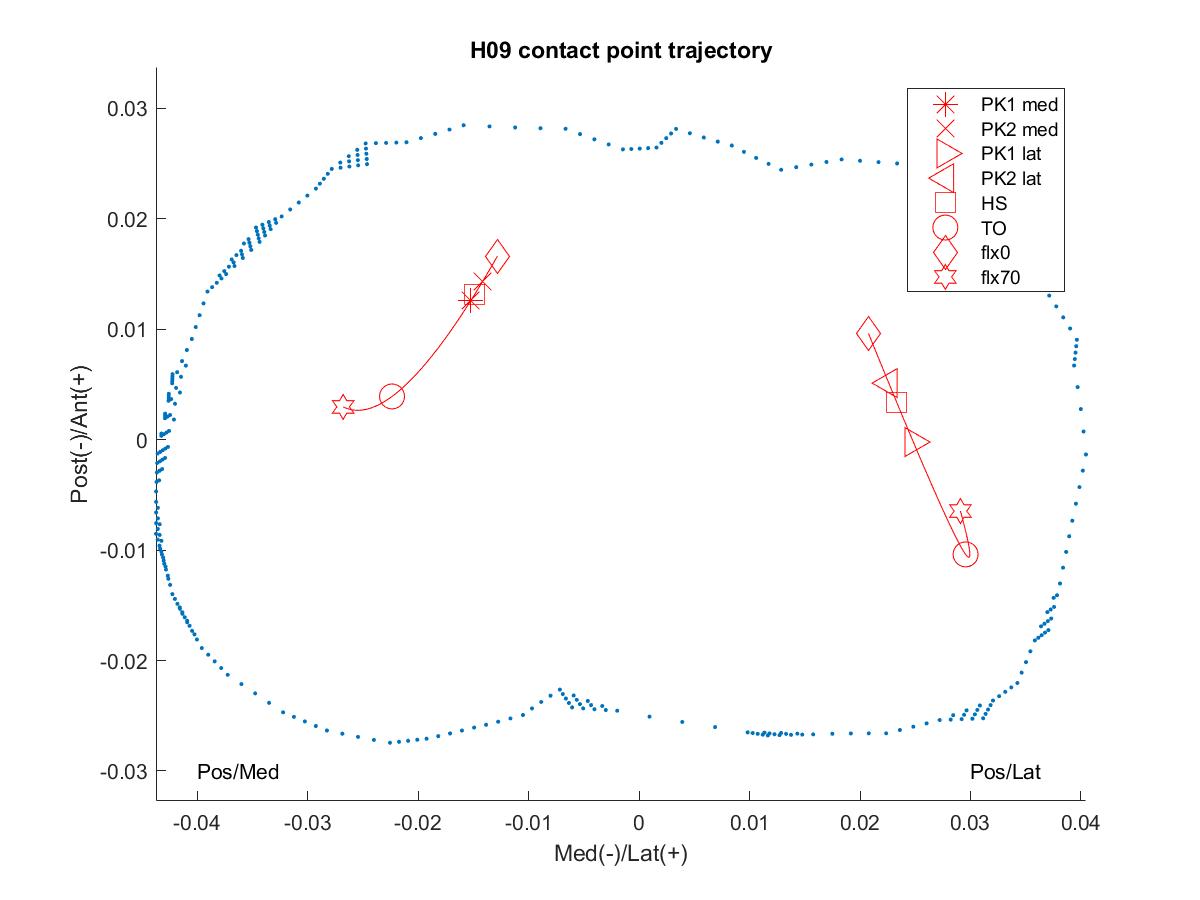

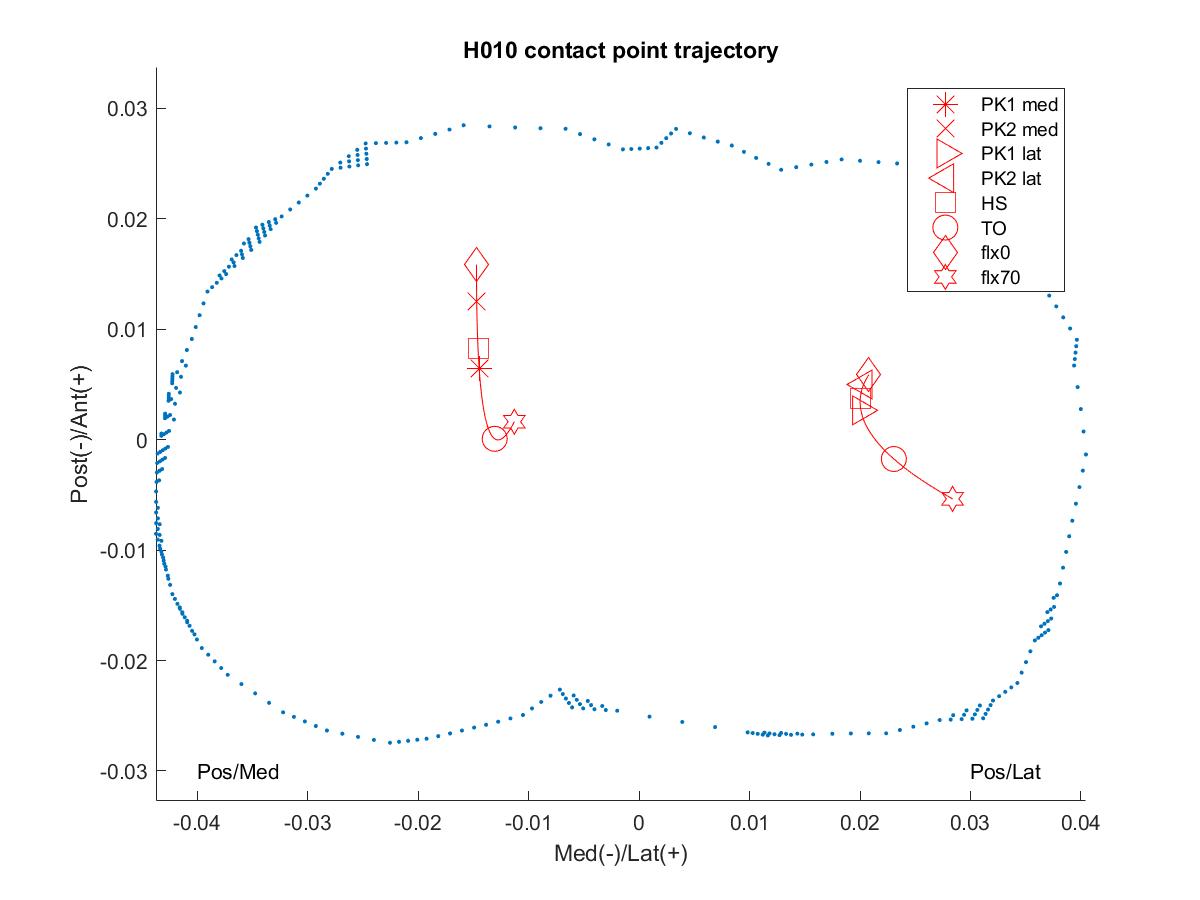

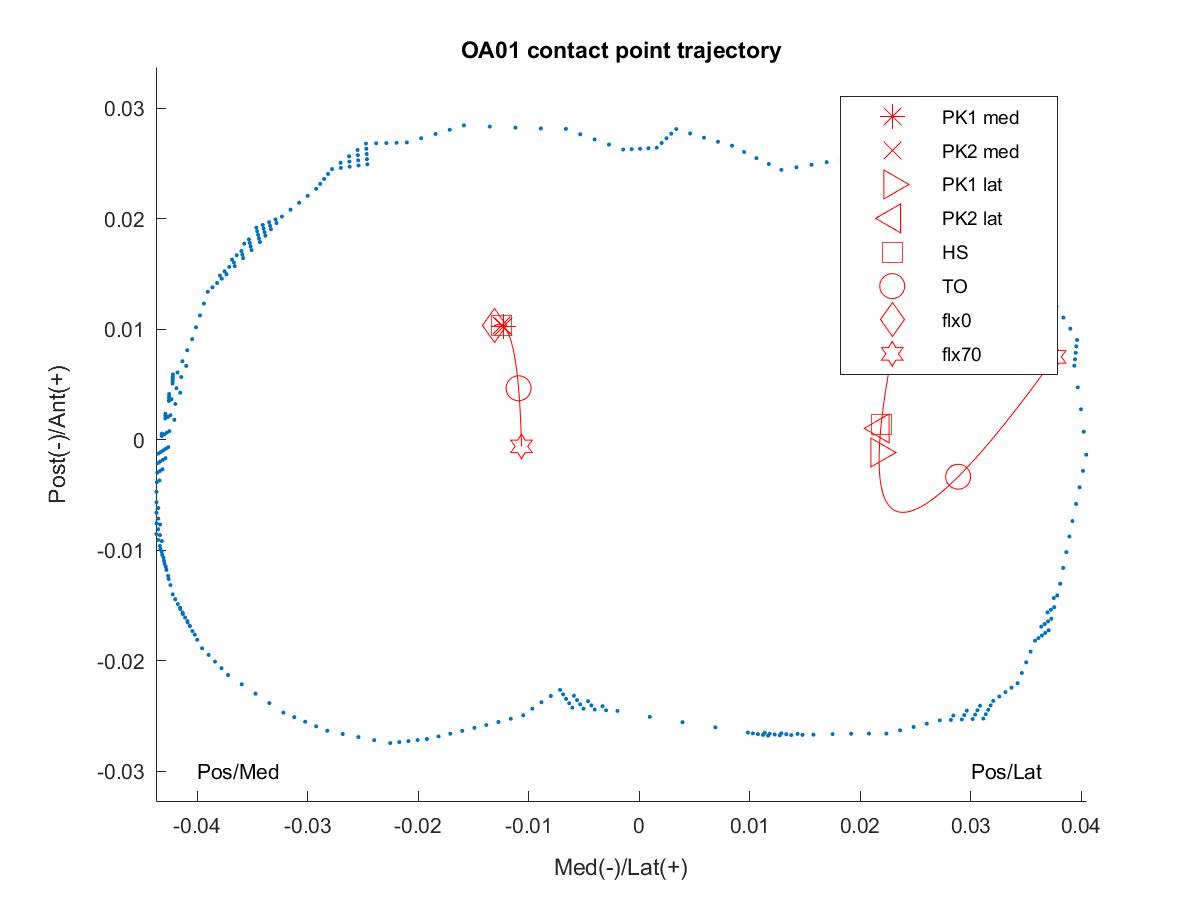

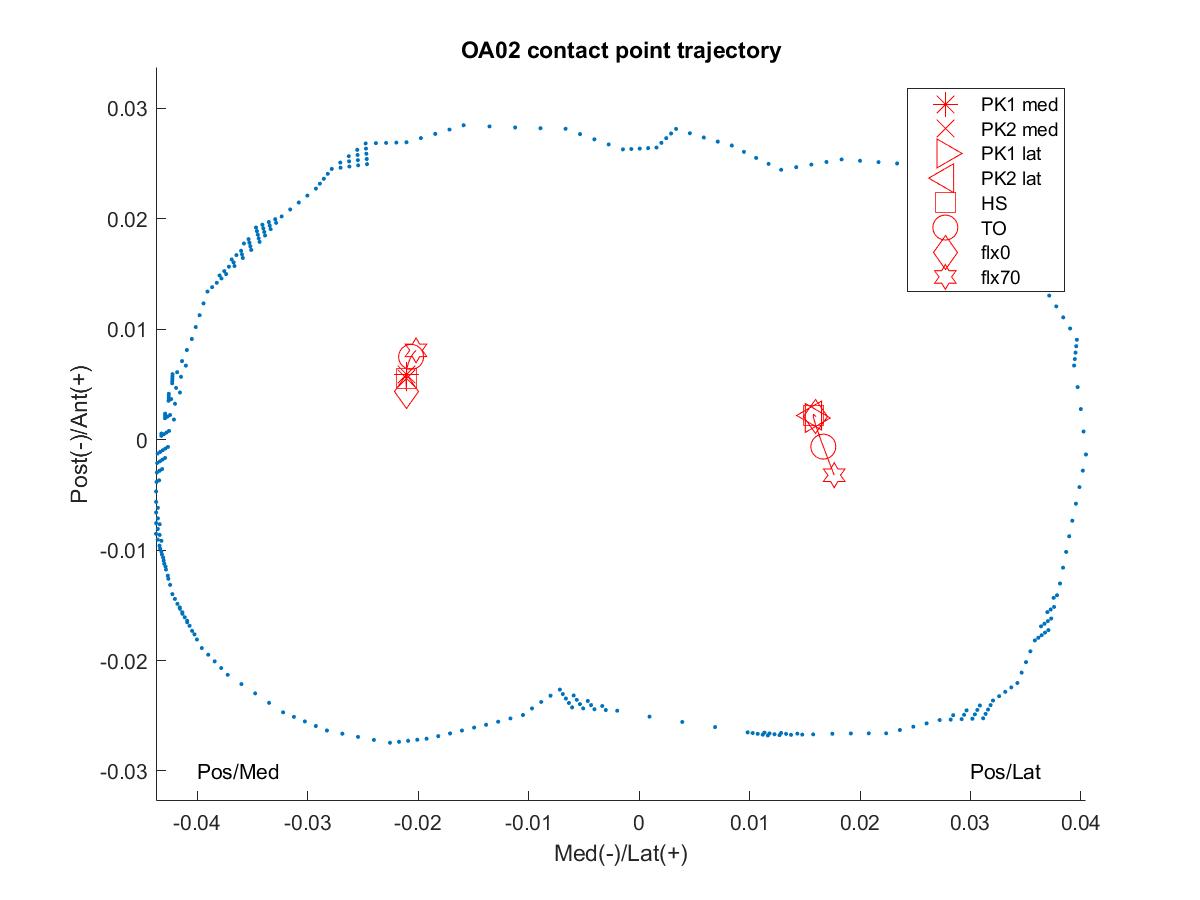

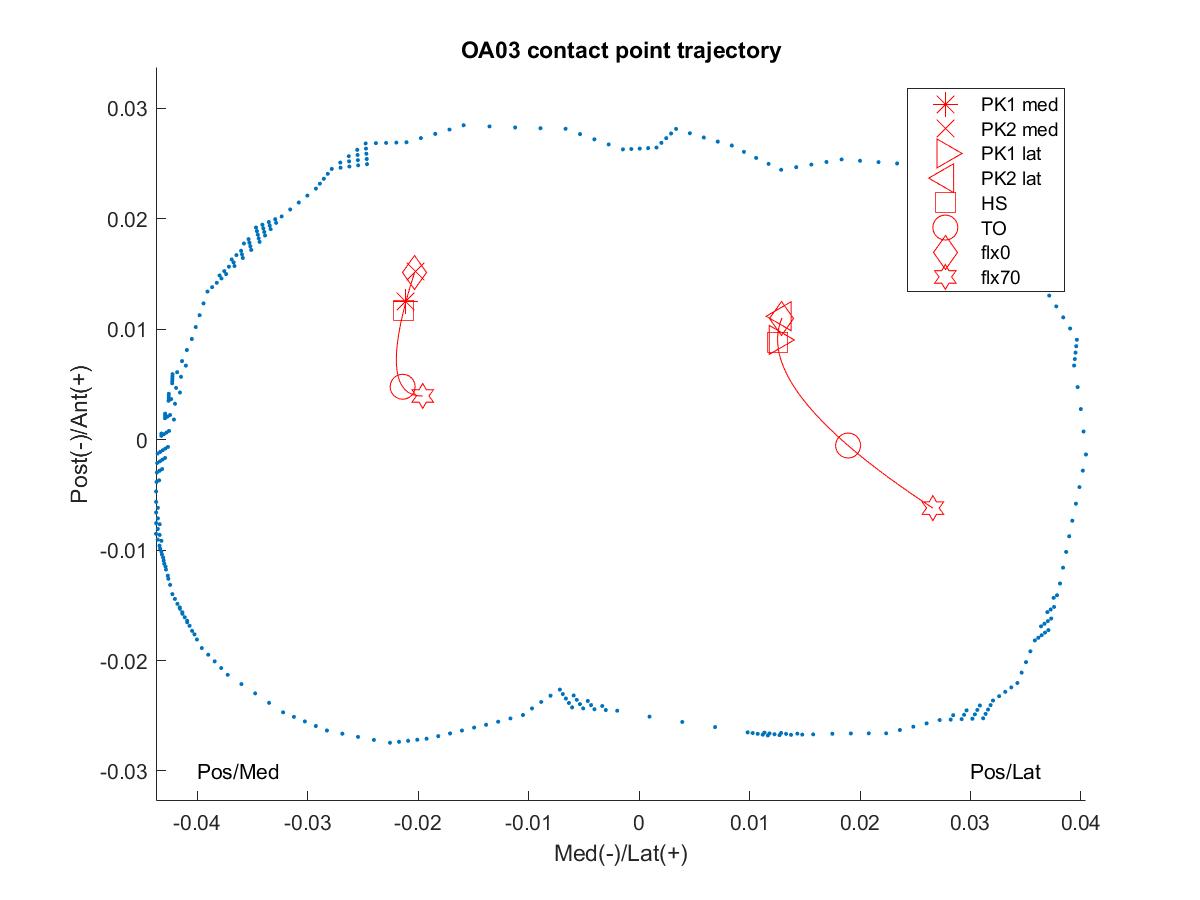

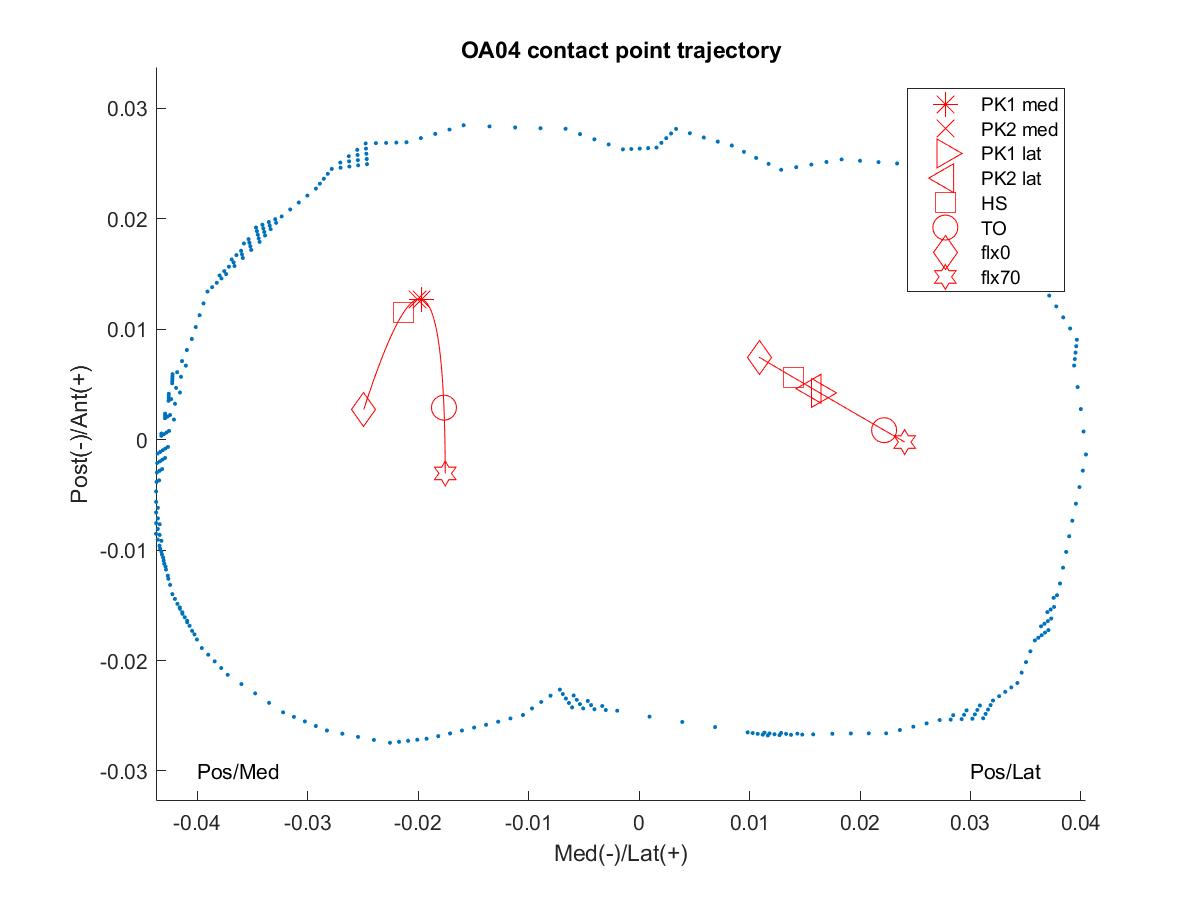

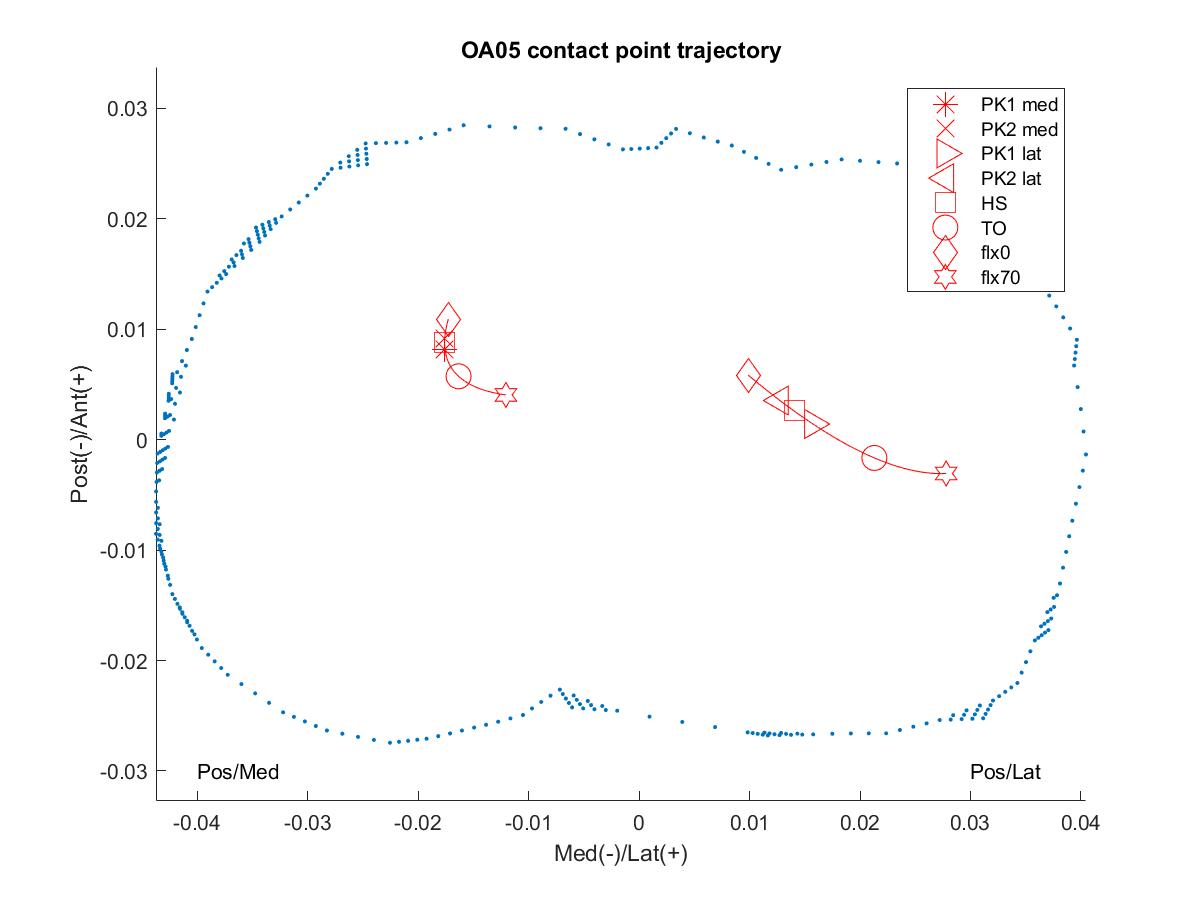

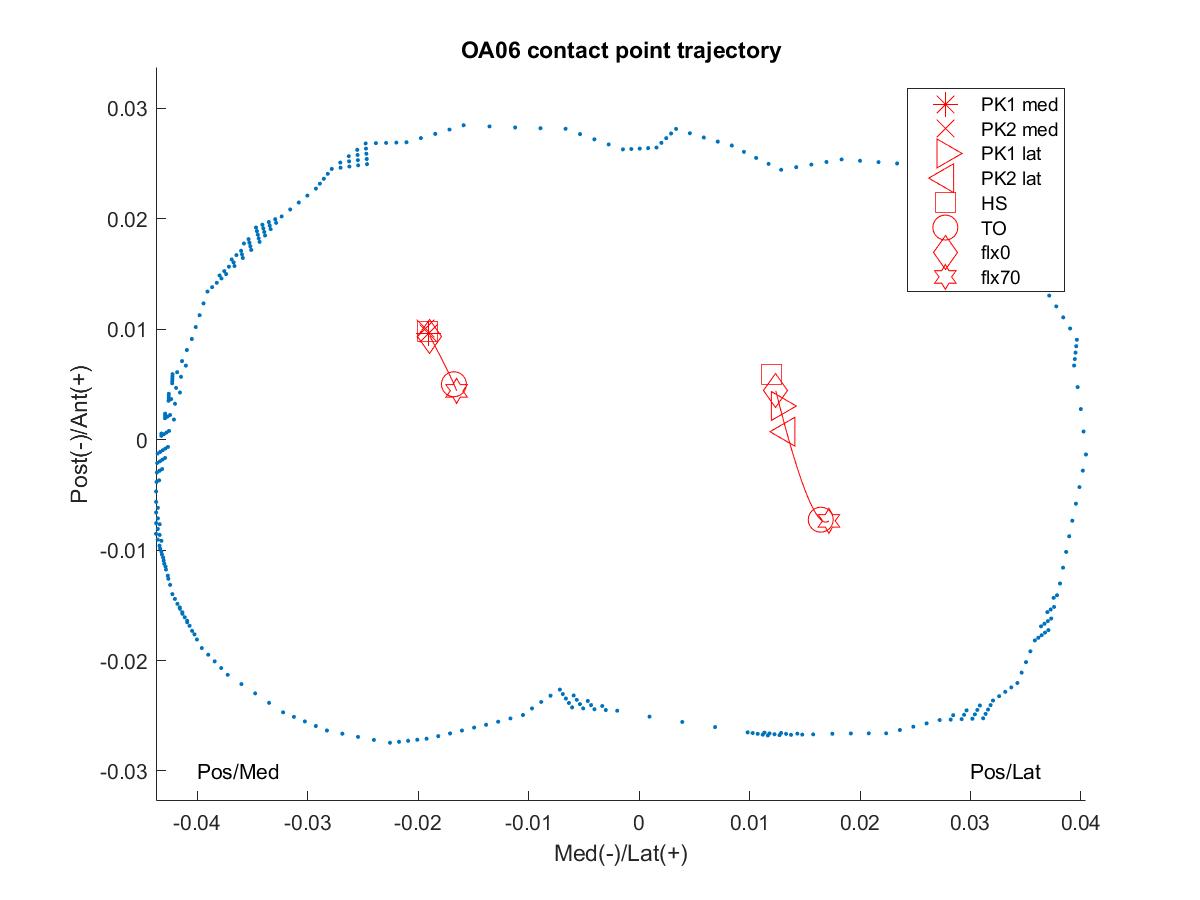

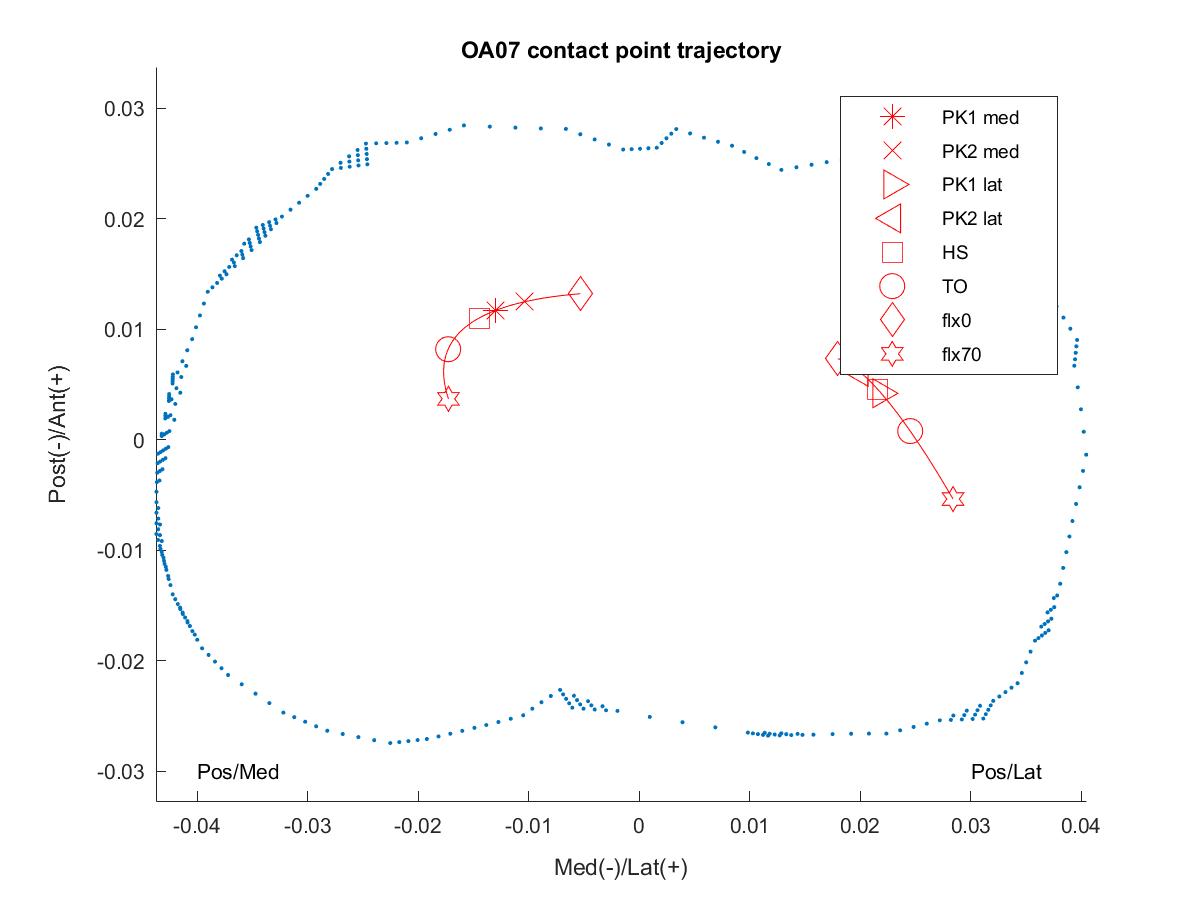

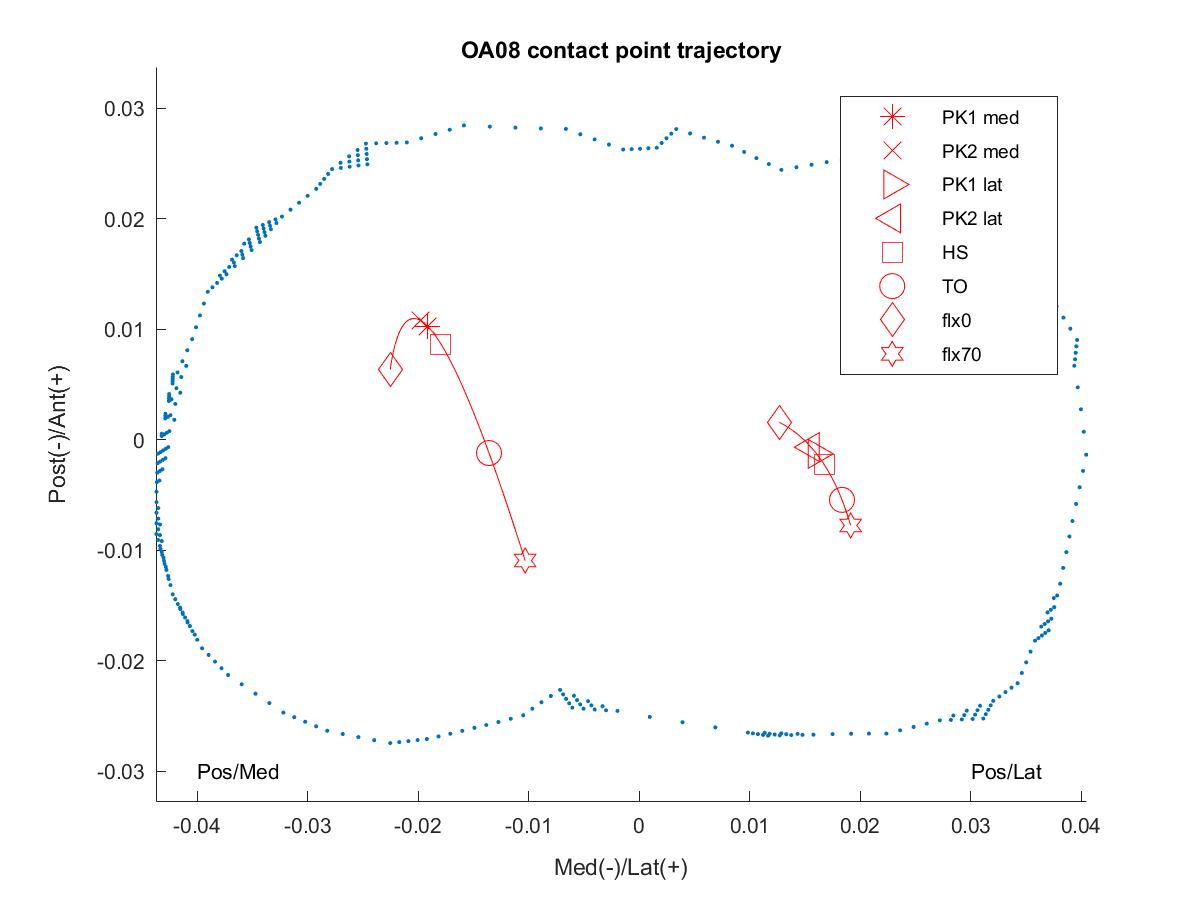

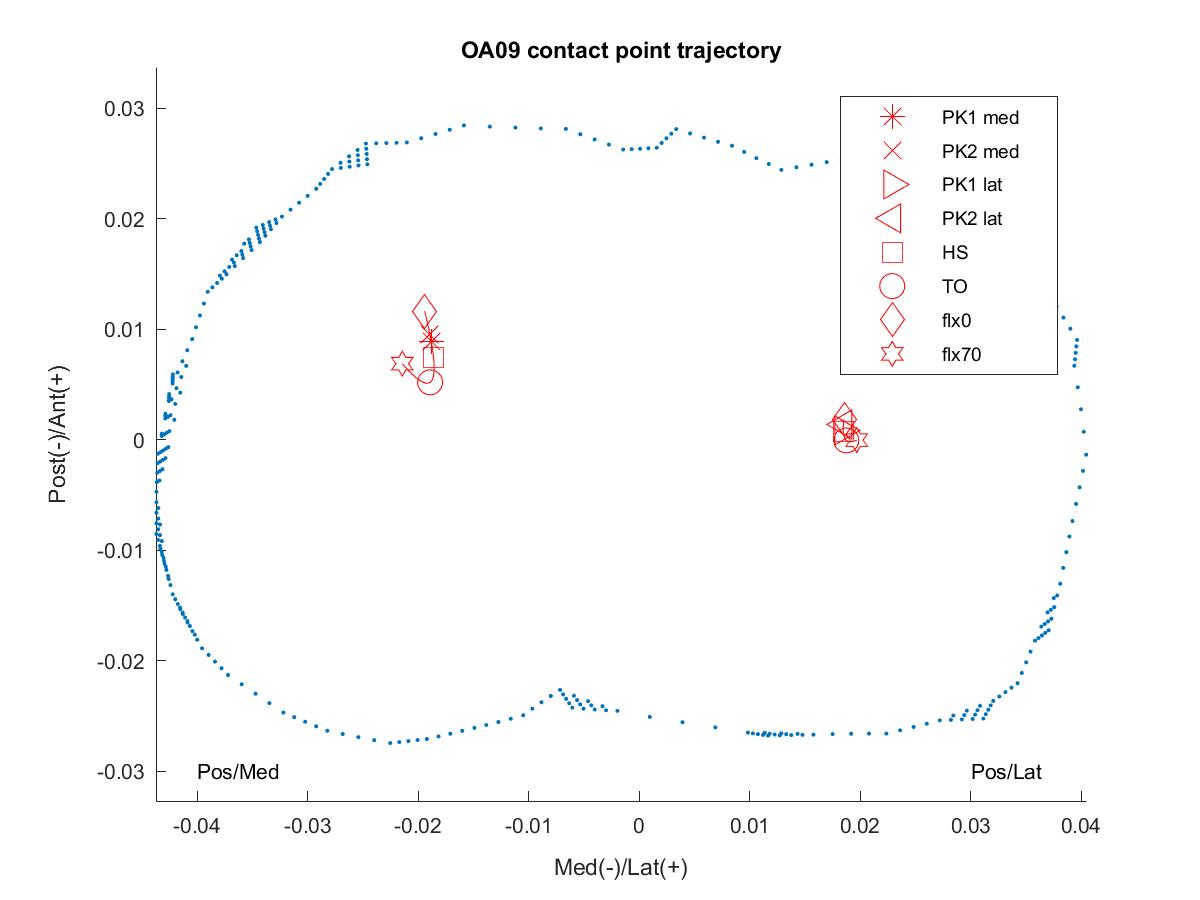

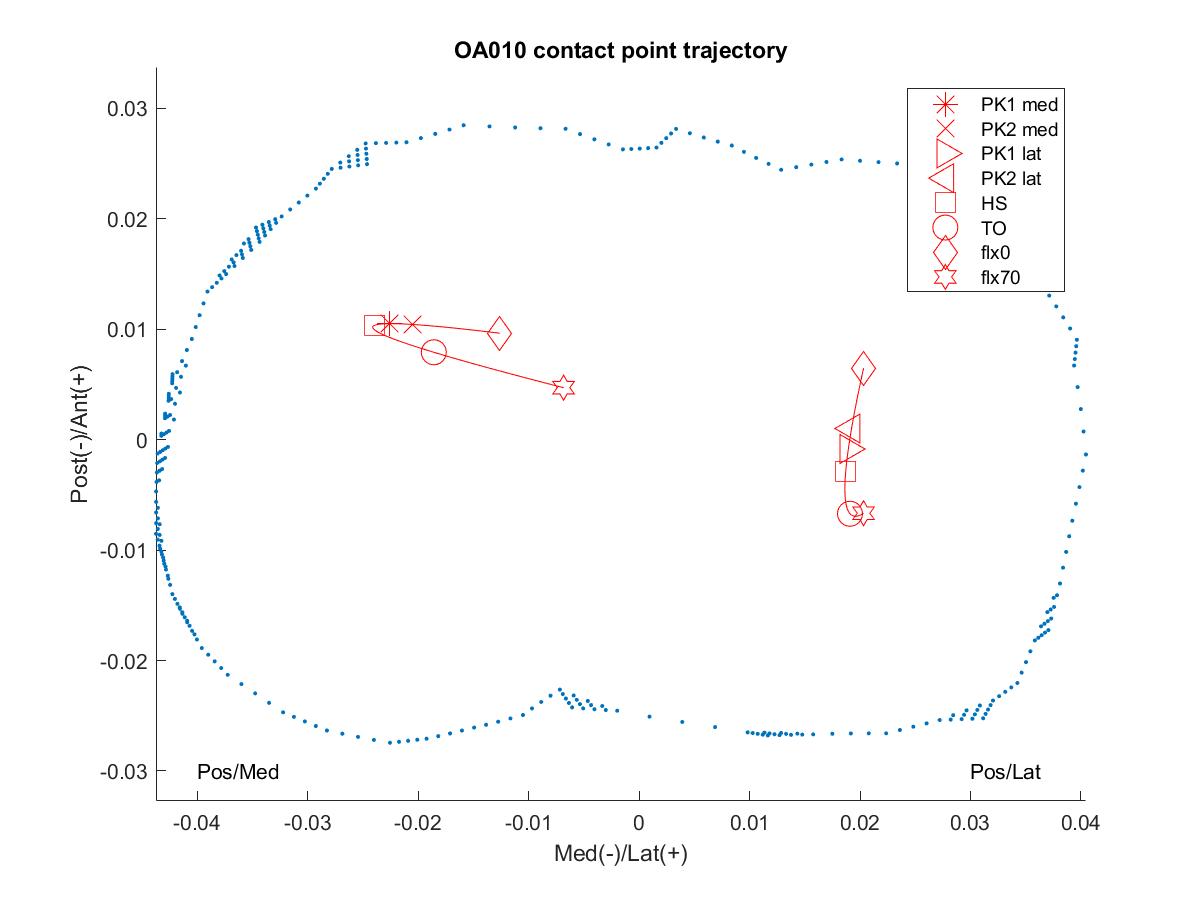

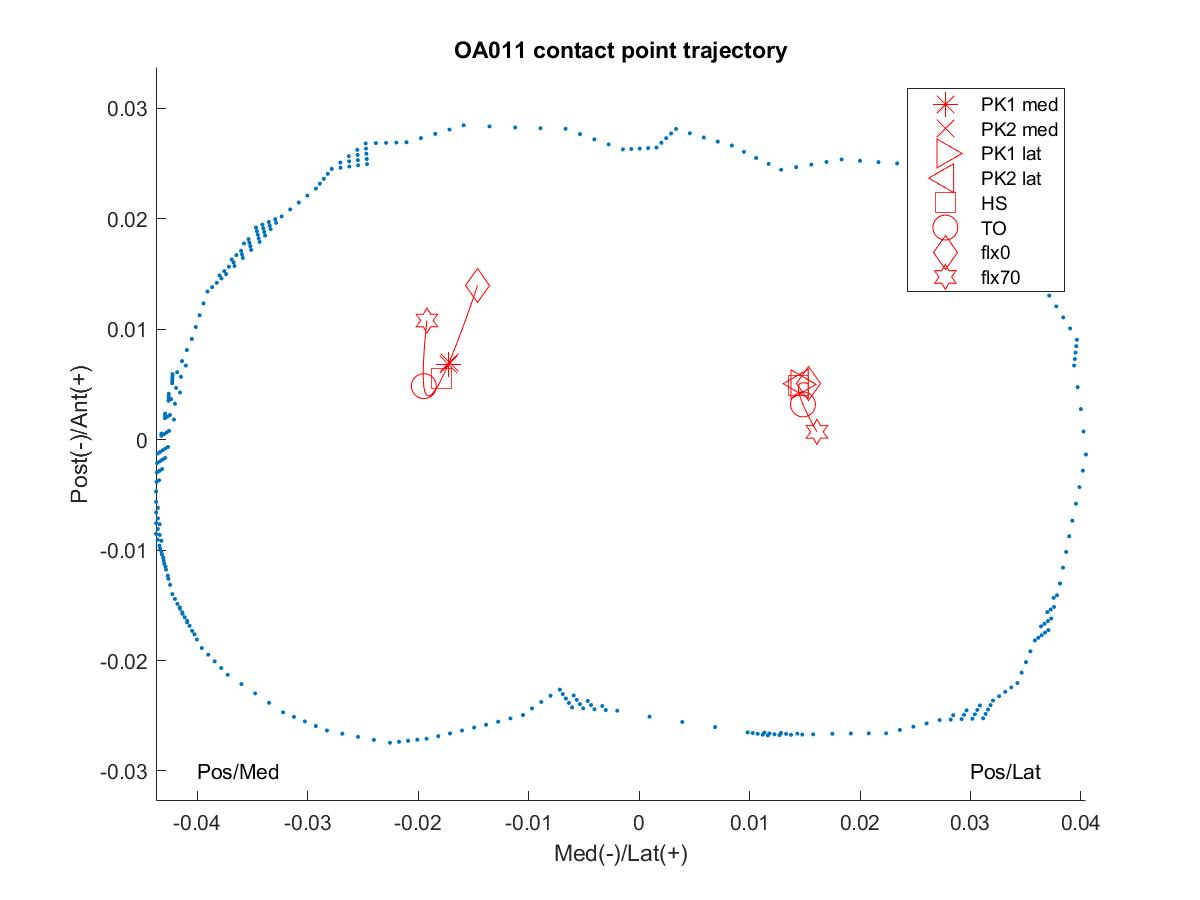

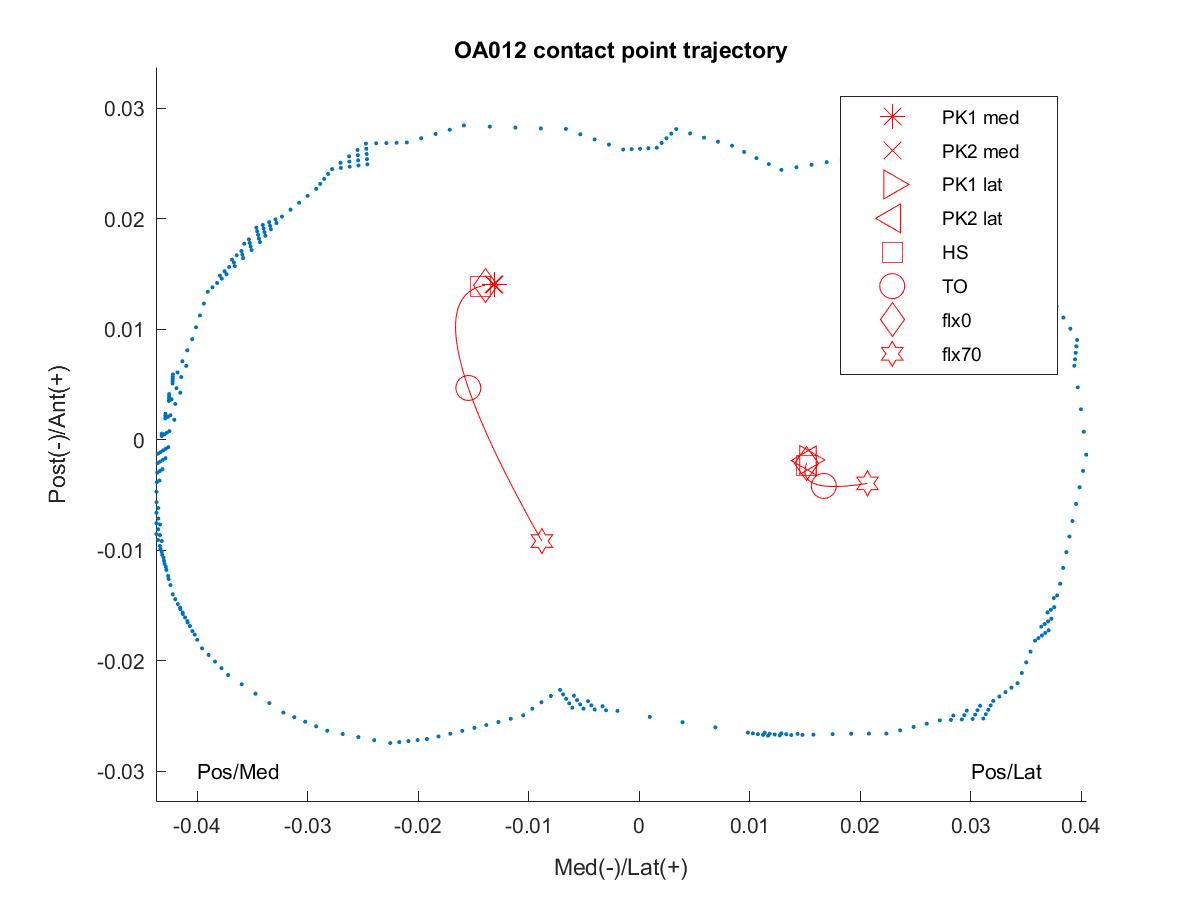

Supplement: Supplementary file 4 — Supplementary Information 4. [file 41598_2021_87978_MOESM4_ESM.docx]
